# Altered angiogenesis as a common mechanism underlying preterm birth, small for gestational age, and stillbirth in women living with HIV

**DOI:** 10.1016/j.ajog.2017.10.003

**Published:** 2017-12

**Authors:** Andrea L. Conroy, Chloe R. McDonald, Joel L. Gamble, Peter Olwoch, Paul Natureeba, Deborah Cohan, Moses R. Kamya, Diane V. Havlir, Grant Dorsey, Kevin C. Kain

**Affiliations:** a Department of Pediatrics, Indiana University School of Medicine, Indianapolis, IN; b SAR Laboratories, Sandra Rotman Center for Global Health, University Health Network–Toronto General Hospital, University of Toronto, Toronto, Canada; c Tropical Disease Unit, Division of Infectious Diseases, Department of Medicine, University of Toronto, Toronto, Canada; d Makerere University–University of California–San Francisco Research Collaboration, Kampala, Uganda; e Makerere University College of Health Sciences, Kampala, Uganda; f Department of Obstetrics and Gynecology, University of California–San Francisco, San Francisco, CA; g HIV/AIDS Division, San Francisco General Hospital, San Francisco, CA

**Keywords:** angiogenesis, HIV-1, placental growth factor, pregnancy, preterm birth, small for gestational age, soluble endoglin, soluble fms-like tyrosine kinase-1, stillbirth

## Abstract

**Background:**

Angiogenic processes in the placenta are critical regulators of fetal growth and impact birth outcomes, but there are limited data documenting these processes in HIV-infected women or women from low-resource settings.

**Objective:**

We sought to determine whether angiogenic factors are associated with adverse birth outcomes in HIV-infected pregnant women started on antiretroviral therapy.

**Study Design:**

This is a secondary analysis of samples collected as part of a clinical trial randomizing pregnant women and adolescents infected with HIV to lopinavir/ritonavir-based (n = 166) or efavirenz-based (n = 160) antiretroviral therapy in Tororo, Uganda. Pregnant women living with HIV were enrolled between 12-28 weeks of gestation. Plasma samples were evaluated for angiogenic biomarkers (angiopoietin-1, angiopoietin-2, vascular endothelial growth factor, soluble fms-like tyrosine kinase-1, placental growth factor, and soluble endoglin) by enzyme-linked immunosorbent assay between: 16-<20, 20-<24, 24-<28, 28-<32, 32-<36, 36-<37 weeks of gestation. The primary outcome was preterm birth.

**Results:**

In all, 1115 plasma samples from 326 pregnant women and adolescents were evaluated. There were no differences in angiogenic factors according to antiretroviral therapy group (*P* > .05 for all). The incidence of adverse birth outcomes was 16.9% for spontaneous preterm births, 25.6% for small-for-gestational-age births, and 2.8% for stillbirth. We used linear mixed effect modelling to evaluate longitudinal changes in angiogenic factor concentrations between birth outcome groups adjusting for gestational age at venipuncture, maternal age, body mass index, gravidity, and the interaction between treatment arm and gestational age. Two angiogenic factors–soluble endoglin and placental growth factor–were associated with adverse birth outcomes. Significantly higher concentrations of soluble endoglin throughout gestation were found in study participants destined to deliver preterm [likelihood ratio test, χ^2^(1) = 12.28, *P* < .0005] and in those destined to have stillbirths [χ^2^(1) = 5.67, *P* < .02]. By contrast, significantly lower concentrations of placental growth factor throughout gestation were found in those destined to have small-for-gestational-age births [χ^2^(1) = 7.89, *P* < .005] and in those destined to have stillbirths [χ^2^(1) = 21.59, *P* < .0001].

**Conclusion:**

An antiangiogenic state in the second or third trimester is associated with adverse birth outcomes, including stillbirth in women and adolescents living with HIV and receiving antiretroviral therapy.

Click Supplemental Materials and Video under article title in Contents at **ajog.org**

## Introduction

It is estimated that 17.8 million of 36.7 million people living with HIV in 2015 were women and girls of reproductive age (>15 years).^1^ Widespread use of combined antiretroviral treatment (ART) has dramatically reduced rates of vertical transmission while improving maternal health and birth outcomes. However, rates of adverse birth outcomes–including preterm birth (PTB), small for gestational age (SGA), and stillbirth–remain higher among women and adolescents living with HIV (WLHIV) receiving ART than HIV-uninfected women.^2^, ^3^, ^4^, ^5^, ^6^, ^7^, ^8^, ^9^, ^10^, ^11^ There are few studies investigating the mechanisms associated with adverse birth outcomes in pregnant WLHIV. As the number of pregnant WLHIV having children increases,^12^ it is important to understand the disease processes underlying adverse birth outcomes that, in turn, may facilitate improved clinical management. Recently, HIV was identified as a risk factor for maternal vascular malperfusion in a population of South African women.^13^

Robust placental function is essential for fetal growth and development. Vasculogenic processes in the first trimester regulate de novo formation and growth of blood vessels. Angiogenic processes beginning in the second trimester induce remodeling of the underlying placental architecture to allow for increased blood flow and surface area for nutrient exchange.^14^, ^15^ Altered expression of angiogenic factors is associated with a number of complications in pregnancy including preeclampsia,^16^, ^17^, ^18^, ^19^, ^20^, ^21^, ^22^, ^23^, ^24^, ^25^, ^26^, ^27^ systemic lupus erythematosus and/or antiphospholipid antibodies,^28^ fetal growth restriction,^22^, ^29^, ^30^ preterm delivery,^31^ and spontaneous abortion/stillbirth,^21^, ^32^, ^33^ suggesting placental stress responses triggered by placental malperfusion can lead to systemic changes in angiogenic factors.^34^, ^35^, ^36^

The vascular endothelial growth factor (VEGF) family of proteins, including placental growth factor (PlGF), are proangiogenic mediators that are synthesized by trophoblast, and endothelial cells of the placental villi. VEGF and PlGF bind VEGF receptor 1 (fms-like tyrosine kinase [Flt]-1) and VEGF binds VEGF receptor 2 on the endothelium to induce vascular proliferation, migration, and sprouting.^14^, ^37^, ^38^ Flt-1 can be alternatively spliced to generate the antiangiogenic protein soluble Flt (sFlt)-1.^39^ The angiopoietins (Ang) bind their cognate receptor, tyrosine-protein kinase Tie-2. Ang-1 induces maturation and stabilization of the vasculature while Ang-2 generally causes destabilization and induces angiogenesis.^15^ Soluble endoglin (sEng) is a soluble receptor of transforming growth factor (TGF)-β that binds TGF-β and reduces its bioavailability.^40^ sEng appears to inhibit the immunoregulatory actions of TGF-β and acts as an antiangiogenic factor in the placenta by inhibiting vascular permeability and nitric-oxide-mediated vasodilation.^40^, ^41^

Angiogenic processes in the placenta affect pregnancy, but there are limited data documenting these processes in the context of HIV infection and low-resource settings where rates of adverse birth outcomes are the highest. We hypothesize that alterations in angiogenic factors are associated with adverse birth outcomes in pregnant WLHIV. To test this hypothesis we longitudinally characterized circulating plasma levels of angiogenic proteins in a cohort of WLHIV initiated on ART.^42^ In this report we describe the kinetics of angiogenic factors over pregnancy, compare angiogenic factors between different ART regimens (efavirenz- vs lopinavir/ritonavir-based ART), and evaluate whether angiogenic factors predict adverse birth outcomes (spontaneous PTB, SGA, and stillbirth).

## Materials and Methods

### Ethics statement

Written informed consent was obtained from all participants. Ethical approval was received from Makerere University School of Medicine (Sept. 20, 2009; reference 2009-141); the Uganda National Council for Science and Technology; the University of California–San Francisco (Aug. 9, 2009; reference 10-02958); and the University Health Network (March 13, 2014; reference 14-7313-AE). This trial was registered: ClinicalTrials.gov (identifier: NCT00993031).

### Study population

Plasma samples were collected from pregnant WLHIV participating in a randomized controlled trial of protease inhibitor vs nonnucleoside reverse transcriptase inhibitor–based ART in Tororo, Uganda, from 2009 through 2013.^42^ Participants were HIV-infected, ≥16 years of age, and pregnant (12-28 weeks of gestation by last menstrual period with confirmation by ultrasound). Eligibility for enrollment was not dependent on CD4 cell count. Patients were ineligible to participate if they had received ART (including any abbreviated monotherapy) or dual therapy with nevirapine in the last 24 months. Subjects received standard antenatal care according to Ugandan Ministry of Health Guidelines (http://www.health.go.ug/docs/ucg_2010.pdf). Blood pressure and urine protein was assessed at enrollment and routine antenatal visits. Participants on protease inhibitor–based ART received lopinavir/ritonavir (n = 166) and those on nonnucleoside reverse transcriptase inhibitor–based ART received efavirenz (n = 160). Socioeconomic status was assessed as described.^43^

### Laboratory assays

Blood collection and laboratory work was performed at baseline and at all subsequent antenatal visits. Clinical tests included complete blood cell count and determination of CD4^+^/CD8^+^ T-lymphocyte subsets. Standardized assessments were completed at delivery including gestational age and birthweight (using an electronic scale).

### Study outcomes

Women were eligible for inclusion in this secondary analysis if they had a singleton pregnancy with known birth outcome, and samples collected within 6 prespecified gestational age bins: 16-<20, 20-<24, 24-<28, 28-<32, 32-<36, 36-<37 weeks of gestation. The primary exposure was biomarker levels and the primary outcome was PTB (so samples were not tested >36 weeks of completed gestation). PTB was defined as delivery <37 weeks of gestation (ultrasound dated), SGA was defined using INTERGROWTH standards,^44^ and stillbirth defined as intrauterine fetal demise ≥20 weeks of gestation.

### Enzyme-linked immunosorbent assays

EDTA plasma samples were collected and stored at −80°C prior to testing. Samples were tested in Uganda using commercially available enzyme-linked immunosorbent assays (Duosets, R&D Systems, Minneapolis, MN) with the following ranges, dilution factors, and intraassay coefficients of variation: Ang-1 (313-20,000 pg/mL, 1:10, 5.6%); Ang-2 (93.8-6000 pg/mL, 1:20, 6.1%); sEng (250-16,000 pg/mL, 1:20, 7.4%); sFlt-1 (250-16,000 pg/mL, 1:5, 11.9%); PlGF (63.0-4000 pg/mL, 1:5, 8.1%), and VEGF (31-2000 pg/mL, 1:2). All testing was performed blinded to group and outcome.

### Statistical analysis

Statistical analysis was performed using STATA v14 (StataCorp, College Station, TX), R v3.2.1^45^ (R Foundation for Statistical Computing), and GraphPad Prism v6 (GraphPad Software Inc, La Jolla, CA) software. Descriptive statistics were calculated as n (%) and median (interquartile range). The χ^2^ and Fisher exact tests were used to compare categorical variables. Linear regression was used to assess whether biomarker levels changed over pregnancy. To assess the effect of gestational age on angiogenic factor concentrations between birth outcome groups, we used the lme4^46^ package in R^45^ to construct linear mixed effects (LME) models with random intercept and random slope, adapting the approach employed by Romero et al.^32^ For each biomarker and outcome, we constructed a null model with 4 fixed effects: the linear effect of gestational age, maternal age, body mass index (BMI), and gravidity. To make the intercept meaningful, the gestational age variable was shifted such that the lowest gestational age in our data set (the baseline samples) would be the intercept’s x-value. We also included the interaction between gestational age and treatment arm, to control for the possibility that the treatment affected the biomarker’s rate of change. Treatment arm was omitted as a main effect to constrain the groups to have the same intercept, as baseline differences between the (randomly allocated) groups would have been due to chance. In 2 additional models, we added the birth outcome as a fixed effect: an additive, no-interaction model and an interaction model. Both models were the same as the null but also included outcome as a main effect; and the interaction model further included the interaction between outcome and gestational age. For none of the biomarkers or birth outcomes did the interaction term significantly improve the model fit (*P* > .05 for all). Therefore, the data favor the more parsimonious additive models. For models in which the groups had different intercepts, the fitted lines did not significantly converge or diverge over time. For random effects, all models included a by-participant intercept and by-participant slope for the effect of gestational age. The biomarker levels were transformed using the natural logarithm to stabilize their variance. Residual plots did not show any apparent deviation from homoscedasticity or normality. Statistical significance was assessed using likelihood ratio (LR) tests, which compared in a stepwise fashion the null model, the additive model, and the interaction model. As the PlGF values vary quadratically over time, we added a quadratic interaction term to the model, which significantly improved the model fit [LR test, χ^2^(1) = 157.15, *P* < .0001].

## Results

### Description of the study population

**Figure 1:**
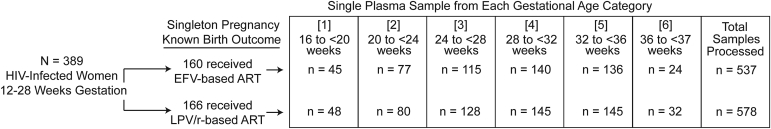
Flow chart of maternal plasma samples processed by gestational age *ART*, antiretroviral therapy; *EFV*, efavirenz; *LPV/r*, lopinavir/ritonavir. *Conroy et al. Angiogenic factors across pregnancy in women living with HIV. Am J Obstet Gynecol 2017*.

A total of 1115 plasma samples were evaluated from 326 women and adolescents (Figure 1). The demographic characteristics of the study population are presented in Table 1. The median age of women was 30 years with a median BMI at enrollment of 21.4 kg/m^2^. The majority of participants (n = 271, 83%) were multigravida. None of the participants were diagnosed with chronic hypertension, preeclampsia, or eclampsia during the study period. Adverse birth outcomes in the cohort were common with 18.1% (n = 59) of participants having a low-birthweight infant (compared to a national estimate of 12%^47^), 16.9% (n = 55) spontaneous PTB, 25.6% (n = 81) SGA, and 2.8% (n = 9) stillborn. The median gestational age of stillbirth deliveries was 31 weeks of gestation. Cause of fetal demise was not ascertained. Malaria infection status and birth outcomes did not differ between participants receiving lopinavir/ritonavir compared to those receiving efavirenz (Supplemental Table 1).

**Table 1:** Descriptive characteristics of study population

	Cohort, n = 326
Demographics	
Age, y	30 (26–33)
BMI, kg/m^2^	21.4 (19.9–23.0)
Socioeconomic status, tertile	
1	111 (35.9)
2	135 (43.7)
3	63 (20.4)
Gestational age at enrollment, wk	23.6 (19.6–27.9)
Previous pregnancies	
0	20 (6.1)
1	35 (10.7)
2	271 (83.1)
Laboratory characteristics	
Hemoglobin level, g/dL	11.0 (10.2–11.8)
White blood cell count, cells/mm^3^	5050 (4200–6200)
Platelet count, ×10^9^/L	210 (173–252)
CD4^+^ T-cell count, cells/mm^3^	369 (271–504)
HIV RNA load, log_10_ copies/mL	4.2 (3.9–4.8)
Delivery characteristics	
Gestational age delivery, wk	38 (37–40)
Birthweight, kg	2890 (2670–3230)
Preterm birth	55 (16.9)
Small for gestational age	81 (25.6)
Stillbirth	9 (2.8)
Placental malaria^a^	24 (8.7)

Continuous variables expressed as median (interquartile range), categorical variables expressed as n (%).

*BMI*, body mass index.

*Conroy et al. Angiogenic factors across pregnancy in women living with HIV. Am J Obstet Gynecol 2017*.

a Defined by positive finding of placental blood smear or polymerase chain reaction.

### Longitudinal changes in angiogenic factors over pregnancy

**Figure 2:**
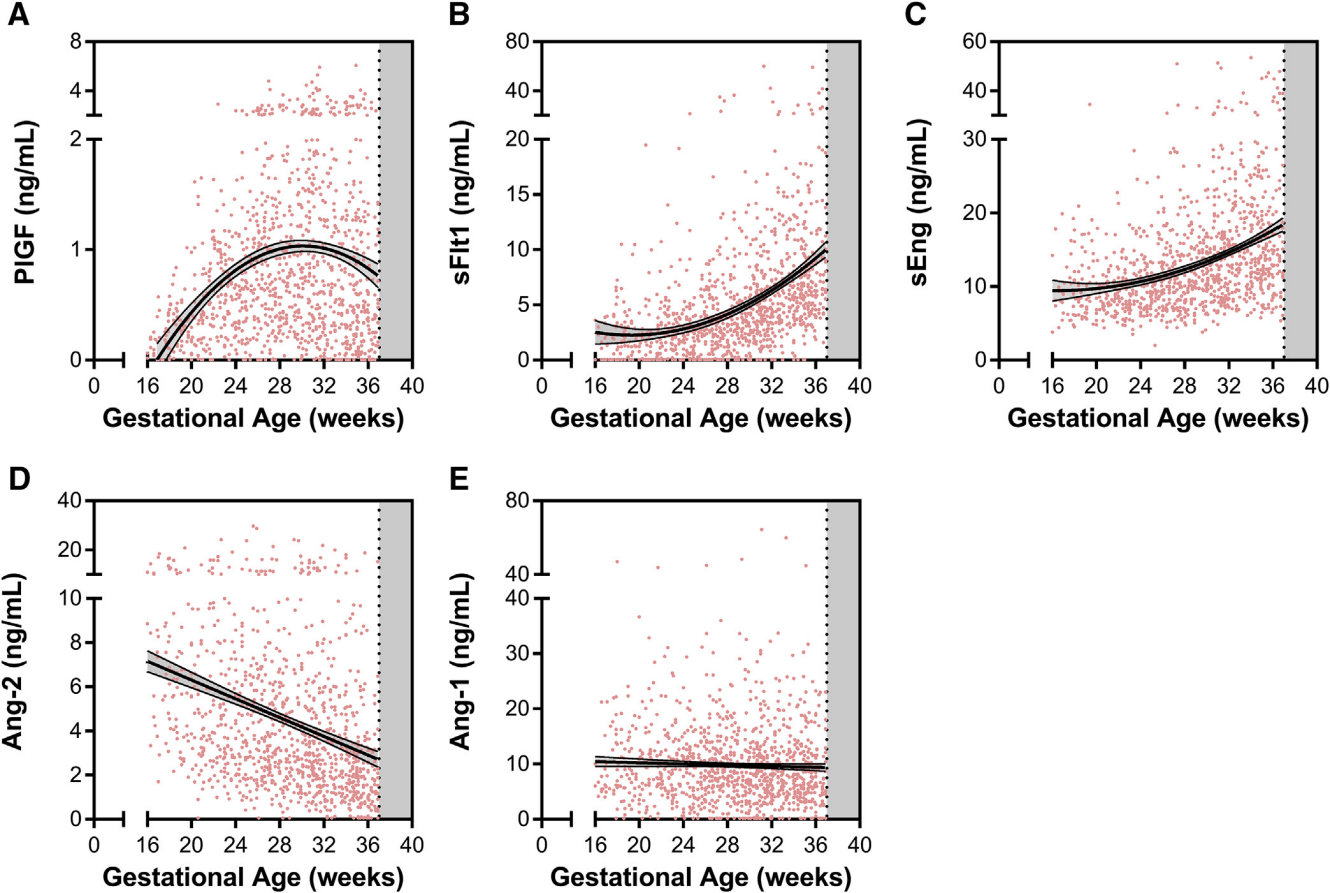
Angiogenic factors in HIV infected study participants receiving antiretroviral therapy Scatter plot of plasma levels of angiogenic factors plotted according to gestational age of sample collection: **A**, placental growth factor (PlGF); **B**, soluble fms-like tyrosine kinase (sFlt)-1; **C**, soluble endoglin (sEng); **D**, angiopoietin (Ang)-2; and **E**, Ang-1. Line indicates best fit line with 95% confidence intervals. *Conroy et al. Angiogenic factors across pregnancy in women living with HIV. Am J Obstet Gynecol 2017*.

**Supplemental Figure 1:**
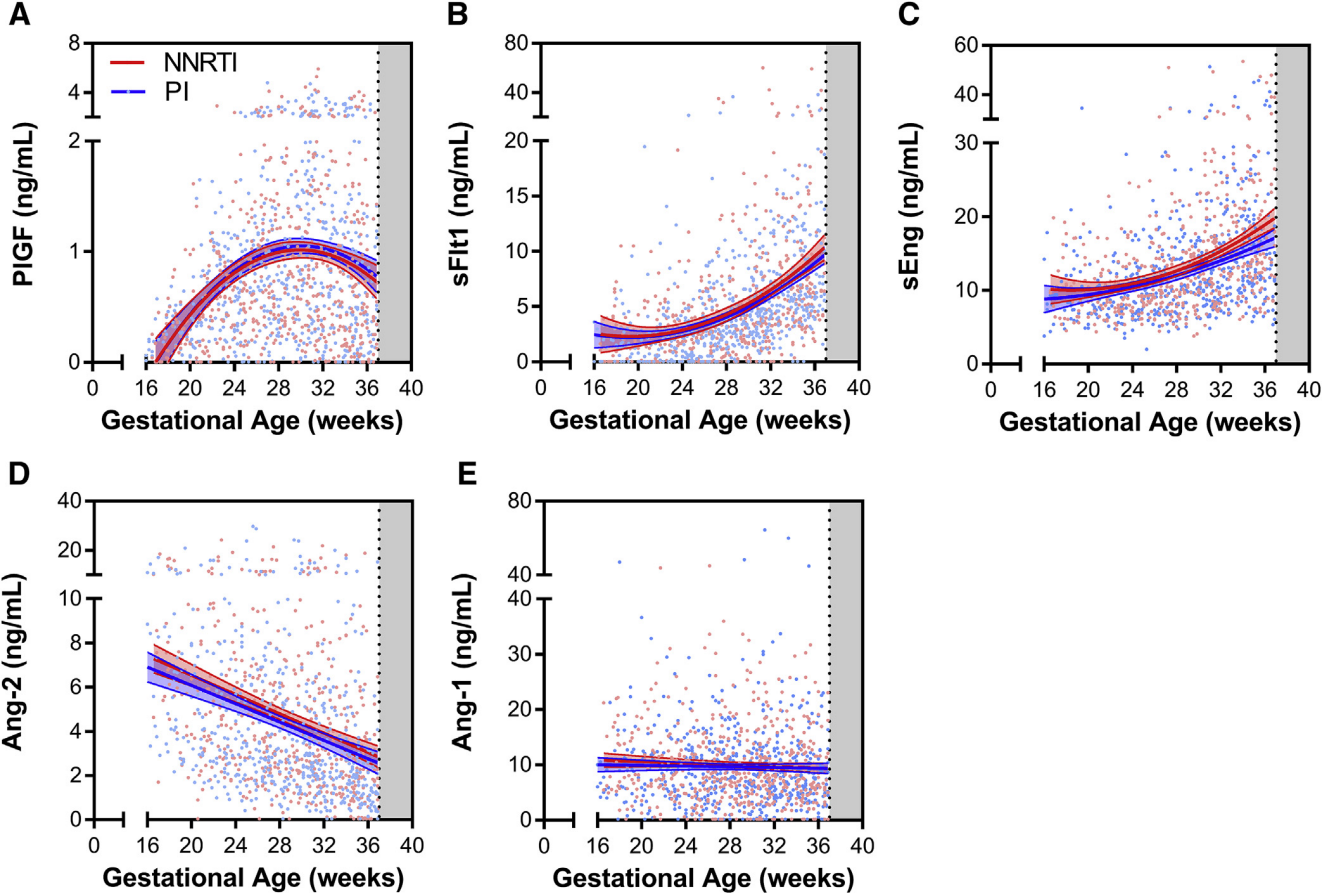
Longitudinal changes in angiogenic factors by treatment arm Scatter plot of plasma levels of angiogenic factors plotted by treatment arm with nonnucleoside reverse transcriptase inhibitor (NNRTI)-based antiretroviral therapy (ART) in red and protease inhibitor (PI)-based ART in blue. Biomarkers plotted according to gestational age of sample collection: **A**, placental growth factor (PlGF); **B**, soluble fms-like tyrosine kinase (sFlt)-1; **C**, soluble endoglin (sEng); **D**, angiopoietin (Ang)-2; and **E**, Ang-1. Line indicates best fit line with 95% confidence intervals. *Conroy et al. Angiogenic factors across pregnancy in women living with HIV. Am J Obstet Gynecol 2017*.

There were no differences in angiogenic proteins by trial arm (Supplemental Figure 1), so subsequent analysis was conducted using the combined cohort. We plotted the longitudinal kinetics of angiogenic factors over pregnancy and observed declining levels of Ang-2 across gestation, and increasing levels PlGF, sFlt-1, and sEng (*P* < .0001 for all) (Figure 2). There were no differences in circulating Ang-1 levels across gestation. Median plasma Ang-2 decreased from 6.6 ng/mL at 16-20 weeks to 2.5 ng/mL by 37 weeks of gestation. Median levels of sEng increased from 9.1-14.7 ng/mL and sFlt-1 increased from 1.9-6.4 ng/mL from 16-20 and 37 weeks of gestation (Figure 2). PlGF levels were 0.24 ng/mL at 16-20 weeks, peaked at 0.84 ng/mL at 28-32 weeks, and declined to 0.40 ng/mL by 37 weeks of gestation (Figure 2). VEGF-A levels were largely undetectable with 87% of samples having concentrations below the bottom standard of 31.3 pg/mL.

### Relationship between angiogenic factors and immune status

To determine whether angiogenic factors were associated with maternal health or immune status, we conducted nonparametric bivariate correlations comparing angiogenic proteins and enrollment laboratory tests. There were no differences between angiogenic proteins measured in the first gestational age bin (16-<20 weeks of gestation) and enrollment hemoglobin, platelet count, viral load, or CD4 count (*P* > .05 for all).

### Relationship between angiogenic factors and birth outcomes

**Figure 3:**
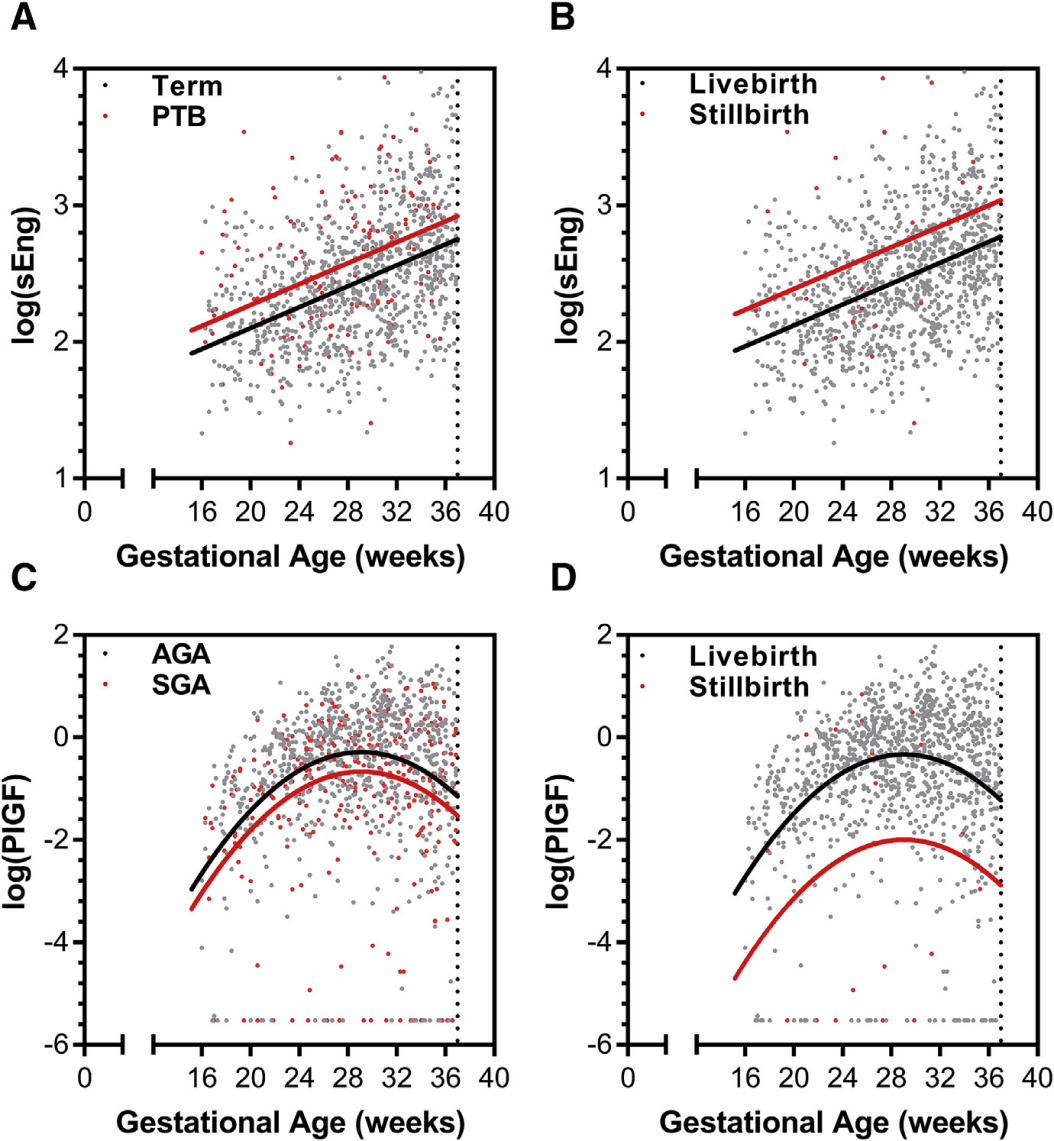
Antiangiogenic shift is associated with adverse birth outcomes in women living with HIV receiving antiretroviral therapy Individual data points colored by birth outcome. Overlaid regression lines are from linear mixed effects models, fitted for subject with average values (conditional on fixed effects only). *AGA*, appropriate for gestational age; *PlGF*, placental growth factor; *PTB*, preterm birth; *sEng*, soluble endoglin; *SGA*, small for gestational age. *Conroy et al. Angiogenic factors across pregnancy in women living with HIV. Am J Obstet Gynecol 2017*.

**Supplemental Figure 2:**
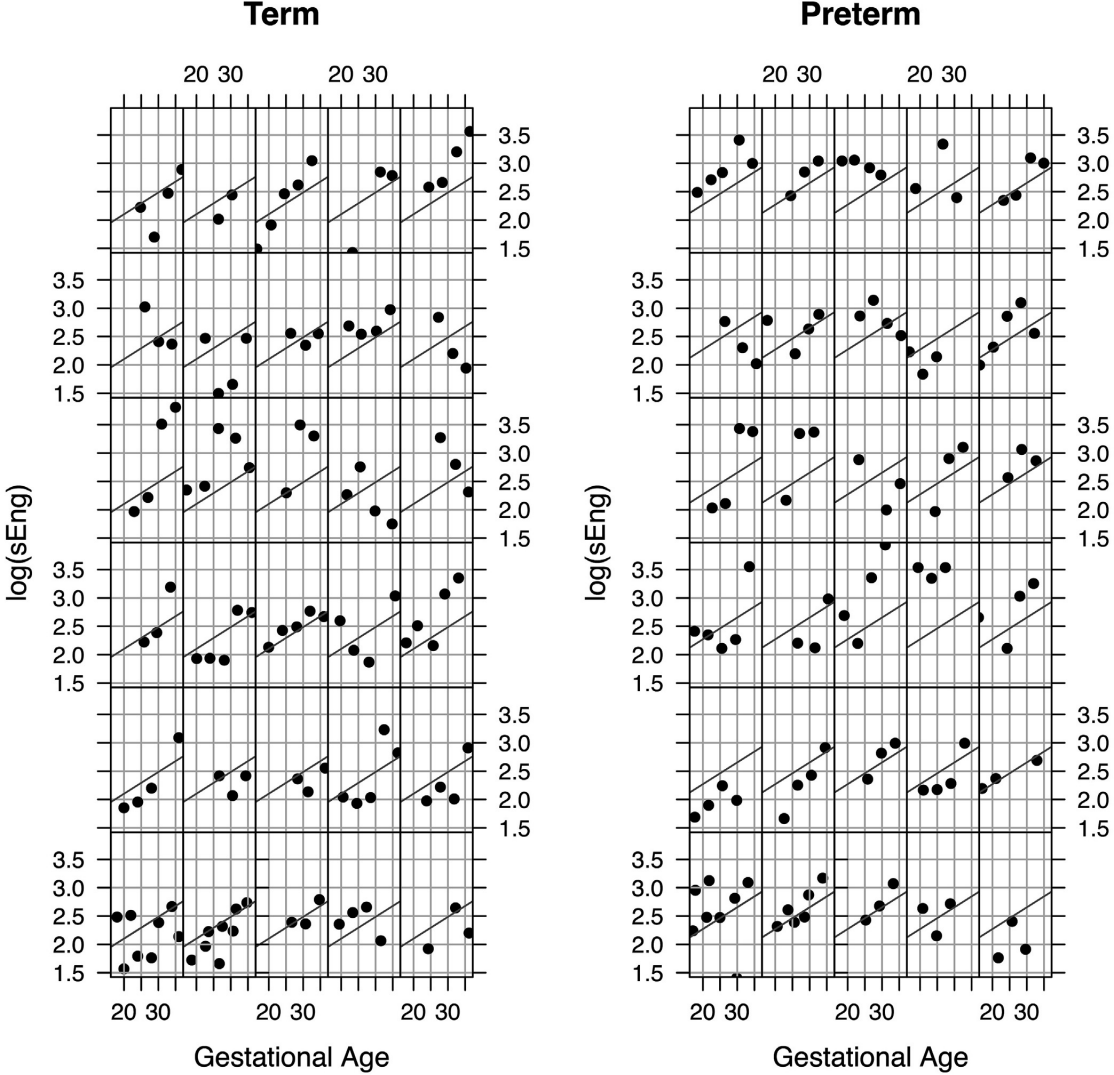
Representative plots of soluble endoglin (sEng) levels over gestation in participants destined to have preterm or term delivery Trellis plots of 60 randomly selected subjects (n = 30 term, n = 30 preterm). Solid line depicts fitted regression line from linear mixed effect model (conditional on fixed effects only). *Conroy et al. Angiogenic factors across pregnancy in women living with HIV. Am J Obstet Gynecol 2017*.

**Supplemental Figure 3:**
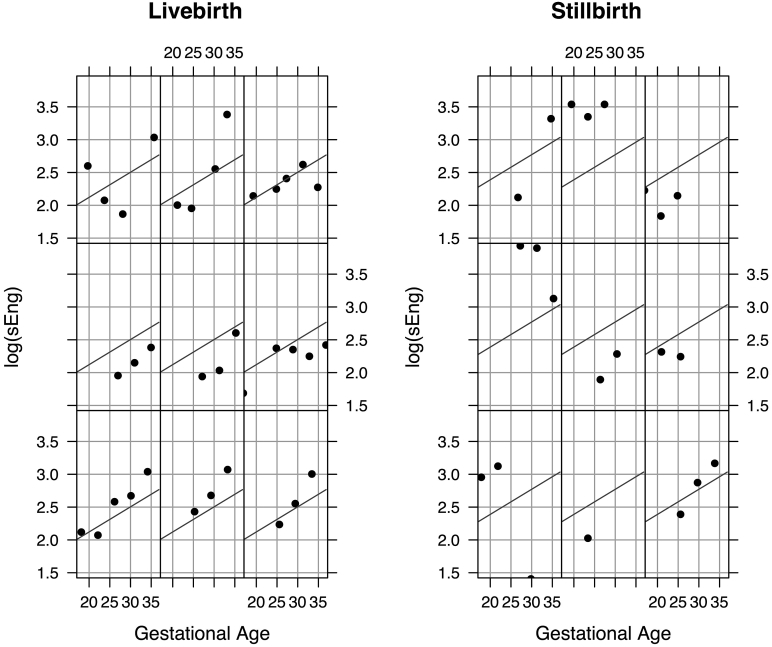
Representative plots of soluble endoglin (sEng) levels over gestation in participants destined to have livebirth or stillbirth infant Trellis plots of 18 randomly selected subjects (n = 9 livebirth, n = 9 stillbirth). Solid line depicts fitted regression line from linear mixed effect model (conditional on fixed effects only). *Conroy et al. Angiogenic factors across pregnancy in women living with HIV. Am J Obstet Gynecol 2017*.

**Supplemental Figure 4:**
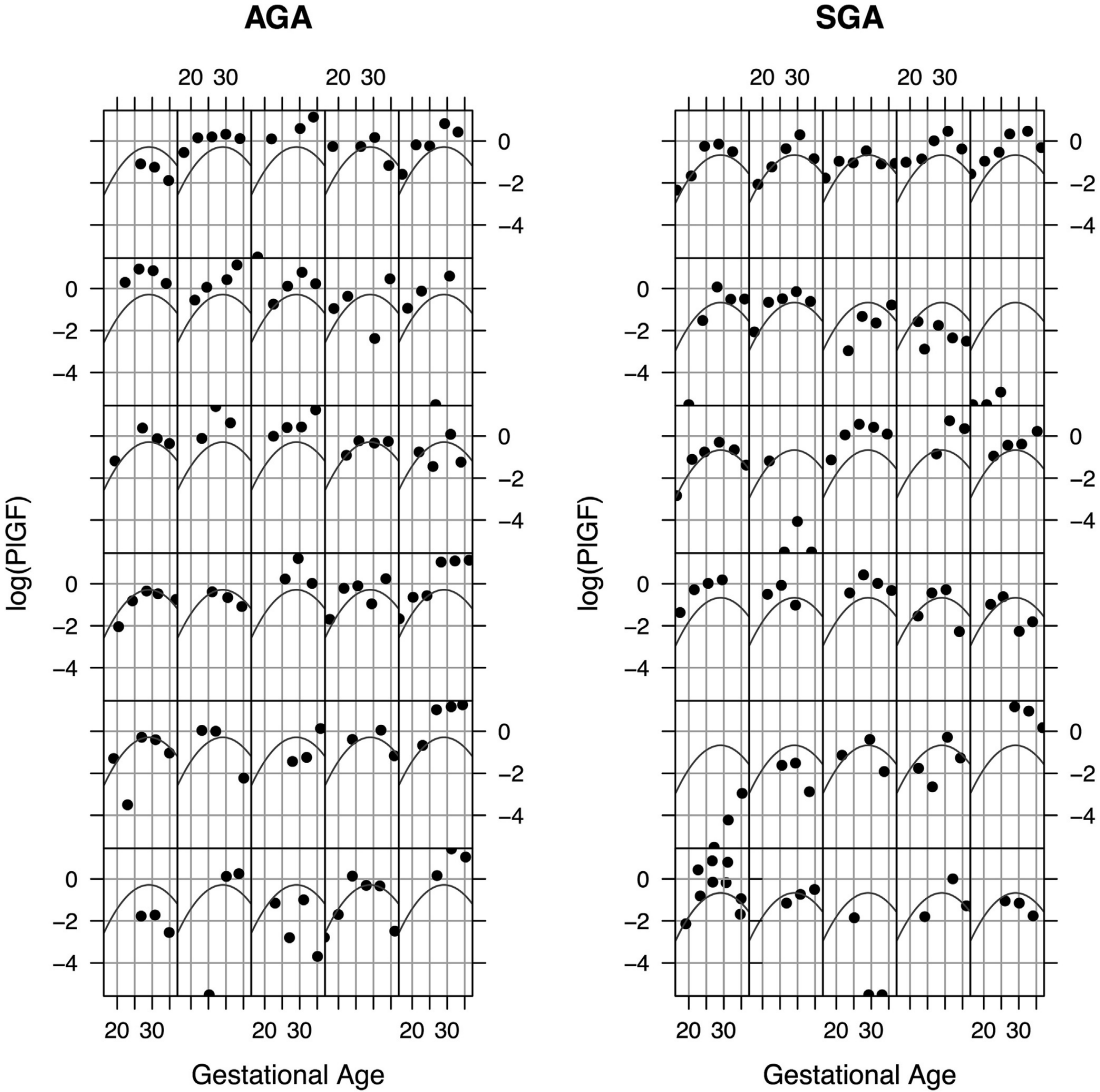
Representative plots of placental growth factor (PlGF) levels over gestation in participants destined to have small-for-gestational-age (SGA) or appropriate-for-gestational-age (AGA) infant Trellis plots of 60 randomly selected subjects (n = 30 AGA, n = 30 SGA). Solid line depicts fitted regression line from linear mixed effect model (conditional on fixed effects only). *Conroy et al. Angiogenic factors across pregnancy in women living with HIV. Am J Obstet Gynecol 2017*.

**Supplemental Figure 5:**
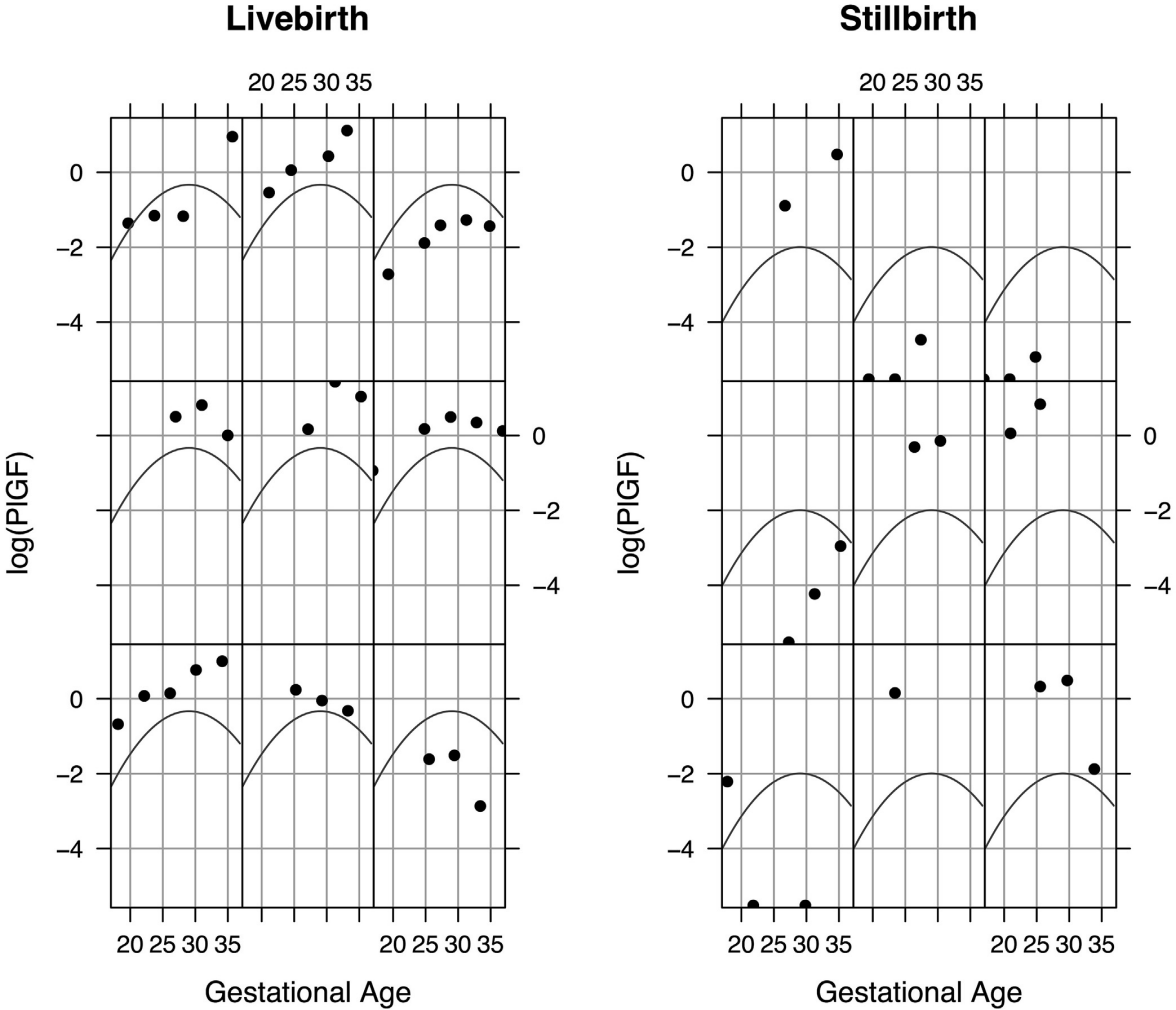
Representative plots of placental growth factor (PlGF) levels over gestation in women destined to have livebirth or stillbirth infant Trellis plots of 18 randomly selected subjects (n = 9 livebirth, n = 9 stillbirth). Solid line depicts fitted regression line from linear mixed effect model (conditional on fixed effects only). *Conroy et al. Angiogenic factors across pregnancy in women living with HIV. Am J Obstet Gynecol 2017*.

LME modeling evaluated the longitudinal changes in angiogenic factor concentrations between birth outcome groups. The models adjusted for gestational age at venipuncture, maternal age, BMI, gravidity, and the interaction between treatment arm and gestational age (Tables 2 and 3, and Table S2, Table S3, Table S4). Two angiogenic factors–sEng and PlGF–were associated with adverse birth outcomes. Significantly higher concentrations of sEng throughout gestation were found in study participants destined to deliver preterm [LR test, χ^2^(1) = 12.28, *P* < .0005] (Figure 3, A) and in those destined to have stillbirths [χ^2^(1) = 5.67, *P* < .02] (Figure 3, B, and Table 2). By contrast, significantly lower concentrations of PlGF throughout gestation were found in those destined to have SGA births [χ^2^(1) = 7.89, *P* < .005] (Figure 3, C) and in those destined to have stillbirths [χ^2^(1) = 21.59, *P* < .0001] (Figure 3, D, and Table 3). To show the natural variability of biomarkers over gestation among individual participants, we generated trellis plots for a random subset of participants with the fitted regression line from the LME model conditional on fixed effects only (Supplemental Figure 2, Supplemental Figure 3, Supplemental Figure 4, Supplemental Figure 5).

**Table 2:** Linear mixed effect modelling of longitudinal changes in soluble endoglin and adverse birth outcomes

	Preterm birth		Stillbirth	
	Beta	SE	Beta	SE
Fixed terms				
(Intercept)	2.13733	0.1760	2.12762	0.1788
Birth outcome	0.16745	0.0475	0.26459	0.1110
Gestational age (shifted)	0.04093	0.0030	0.04064	0.0030
Maternal age	–0.00102	0.0047	–0.00154	0.0048
Enrollment BMI	–0.00455	0.0063	–0.00297	0.0064
Gravidity	–0.01270	0.0109	–0.01047	0.0111
Gestational age: treatment arm interaction	–0.00507	0.0025	–0.00471	0.0026
No. of subjects	320		320	
Observations	1085		1085	
LR test against null model	χ^2^(1) = 12.282, *P* < .0005		χ^2^(1) = 5.6717, *P* < .02	

Linear mixed effect modelling evaluated longitudinal changes in angiogenic factor concentrations between birth outcome groups. Models adjusted for gestational age at venipuncture, maternal age, BMI, gravidity, and interaction between treatment arm and gestational age.

*BMI*, body mass index; *LR*, likelihood ratio.

*Conroy et al. Angiogenic factors across pregnancy in women living with HIV. Am J Obstet Gynecol 2017*.

**Table 3:** Linear mixed effect modelling longitudinal changes in placental growth factor and adverse birth outcomes

	Small for gestational age		Stillbirth	
	Beta	SE	Beta	SE
Fixed terms				
(Intercept)	–3.03817	0.6134	–3.14340	0.5889
Birth outcome	–0.38094	0.1357	–1.66380	0.3520
Gestational age (shifted)	0.36286	0.0264	0.36828	0.0259
Gestational age (shifted) squared	–0.01379	0.0010	–0.01405	0.0010
Maternal age	0.01062	0.0163	0.00881	0.0156
Enrollment BMI	–0.00981	0.0218	–0.00876	0.0208
Gravidity	0.06139	0.0379	0.07530	0.0359
Gestational age: treatment arm interaction	–0.00384	0.0087	–0.00421	0.0085
No. of subjects	311		320	
Observations	1080		1104	
LR test against null model	χ^2^(1) = 7.892, *P* < .005		χ^2^(1) = 21.595, *P* < .0001	

Linear mixed effect modelling evaluated longitudinal changes in angiogenic factor concentrations between birth outcome groups. Models adjusted for gestational age at venipuncture, maternal age, BMI, gravidity, and interaction between treatment arm and gestational age.

*BMI*, body mass index; *LR*, likelihood ratio.

*Conroy et al. Angiogenic factors across pregnancy in women living with HIV. Am J Obstet Gynecol 2017*.

## Comment

### Principal findings of the study

Angiogenic processes in the placenta are critical regulators of fetal growth, but there are limited data documenting these processes in low-resource settings where maternal infections are common. In this study we assessed longitudinal concentrations in angiogenic proteins in HIV-infected pregnant women and adolescents started on ART. There were no differences in angiogenic factors by treatment arm. All proteins except Ang-1 and VEGF-A were dynamically regulated over gestation. Ang-2 levels declined, while sFlt-1 and sEng increased over gestation, and PlGF increased up to 32 weeks of gestation and then declined. Altered expression of angiogenic factors was associated with spontaneous PTB, SGA, and stillbirth. These data suggest an early shift toward an antiangiogenic state is a common pathway associated with adverse birth outcomes in WLHIV, consistent with data from HIV-uninfected women.^22^, ^31^, ^32^, ^34^, ^48^, ^49^, ^50^ Placental malaria was uncommon in this study due to the distribution of insecticide-treated bed nets and daily prophylaxis with trimethoprim-sulfamethoxazole.

### Comparison with previous studies

We evaluated 1115 plasma samples collected from 326 women and adolescents between 16-<37 weeks of gestation. We compared our findings directly to results from women enrolled in a clinical trial receiving calcium supplementation in pregnancy with longitudinal assessment of sFlt-1, PlGF and VEGF,^16^ and sEng^19^ between 8-42 weeks of gestation.^51^ Both sFlt-1 and sEng levels increased over pregnancy starting at 24-28 weeks of gestation. While levels of sFlt-1 increased 3-fold over the third trimester, levels of sEng reached a plateau by 32-26 weeks of gestation. PlGF levels increased in early pregnancy, peaked between 28-32 weeks of gestation, and declined. Our results were consistent with those from the calcium trial suggesting temporal regulation of angiogenic factors is tightly controlled across pregnancy. In both studies, VEGF was measured but was undetectable in the majority of samples.

Despite their important regulatory role in placental vascular development, data on Ang kinetics in pregnancy are limited. Our observations are consistent with a report of decreased placental Ang-2 messenger RNA over pregnancy.^14^ Placental expression of Ang-2 messenger RNA is strongly correlated with circulating Ang-2 protein levels.^14^ Our results are further supported by a study examining plasma Ang levels between 10-37 weeks of gestation where Ang-2 levels decreased and Ang-1 remained the same across gestation.^52^ Collectively these data demonstrate the Ang-Tie-2 axis is dynamically regulated over pregnancy.

There are limited data on the impact of maternal HIV infection on expression of angiogenic factors. Lower PlGF levels have been reported in pregnant WLHIV in South Africa^53^; however, the sample size was small (n = 27 HIV-uninfected, n = 31 WLHIV), and samples were collected a week later in WLHIV. Other studies examining angiogenic factors in nonpregnant individuals infected with HIV have shown increases in the Ang-2:Ang-1 ratio associated with acute HIV infection, and decreased Ang-1 in chronic disease.^54^ In HIV-infected Kenyan women with advanced infection, Ang-2 levels decreased and Ang-1 levels increased following the initiation of ART.^55^ On a cellular level, HIV-infected cells release transactivator of transcription, which accumulates on and is taken up by endothelial cells where it acts in synergy with VEGF-A to modify the cytoskeletal structure of endothelium.^56^, ^57^

There have been a number of studies investigating the relationship between placental angiogenesis, vascular remodeling, and stillbirth. In a study of 22 unexplained stillbirths and 44 age-matched live-born controls, stillbirth was associated with increased placental microvascular density, vasculopathy, and increased vascular permeability,^58^ suggesting increased vascular remodeling in terminal stillbirth placentae. In contrast, in 1269 singleton women with samples collected between 30-34 weeks of gestation, a low PlGF/sFlt-1 ratio was associated with stillbirth.^21^ Levels of sFlt-1 and sEng in the highest quartile of amniotic fluid were associated with increased odds of stillbirth in women with unexplained fetal death.^23^ In a longitudinal nested case-control study of women with a fetal death compared to those with an appropriate-for-gestational-age term delivery, the first trimester (weeks 7-11) was characterized by a proangiogenic phenotype (low sFlt-1, low sEng, high PlGF) and this shifted in favor of an antiangiogenic phenotype over the second and third trimester (between 23-41 weeks of gestation).^32^ Another cohort showed low sFlt-1 and PlGF levels in the first trimester were associated with spontaneous abortion.^33^ Our data support and extend the hypothesis that an antiangiogenic state in the second and third trimester is associated with subsequent stillbirth with lower PlGF and higher sEng levels. As the placental vascular network undergoes continual growth and remodeling, it requires the coordinated regulation of angiogenic factors in a spatial, temporal, and quantitative manner. Additional studies are needed to assess how temporal changes in angiogenic factors during pregnancy relate to the vascular phenotype observed in the placenta at delivery.

Beginning in the second trimester the placenta undergoes a continuous process of sprouting angiogenesis, intercalated growth, and intussusception. This remodeling allows for increased placental volume and surface area for nutrient exchange to support the rapidly growing fetus. Dysregulation of the pathways that mediate these processes midpregnancy may result in preterm delivery or fetal growth restriction if the placenta cannot support this rapid growth. Relative increases in sEng levels across gestation were associated with spontaneous PTB in this cohort, supporting the hypothesis that an antiangiogenic environment can contribute to premature birth.^48^, ^49^ Likewise, reduced PlGF across gestation was associated with SGA, consistent with previous literature linking PlGF to placental and fetal growth and development.^30^, ^50^

### Strengths and weaknesses

This study has several strengths including ultrasound-confirmed gestational dating and frequent blood sampling. Despite routine clinical monitoring over pregnancy including monthly blood pressure and proteinuria assessments, no women in this cohort developed preeclampsia. This study is the first to present detailed kinetics data from a low-resource setting where the burden of disease is greatest, but for which we have the least amount of data. The lack of an HIV-uninfected comparison group in this study is a limitation that prevents us from discussing the generalizability of the findings or the relative impact of maternal HIV infection. While we did not observe any changes in angiogenic factor expression by treatment arm, CD4 count, or HIV-1 RNA viral load at enrollment, additional studies are needed to delineate the role of HIV-1 infection on the expression of angiogenic factors to determine whether infection itself modifies also the risk of adverse birth outcomes through dysregulated angiogenesis.

### Research and clinical implications

This study was conducted in a rural area of southeastern Uganda where the low incidence of preeclampsia is consistent with clinical reports from the region. While the reason for this is unknown, we speculate that a relative absence of risk factors for preeclampsia, including nulliparity, coupled with lower weight gain over gestation contributed to a lower risk of preeclampsia.^59^ The median weekly weight gain in the study was 0.2 kg with nearly 20% of women gaining no weight over the study period.^60^ Additional studies are needed to validate these findings in HIV-infected women and in populations where preeclampsia is more common to ascertain the generalizability of these findings, and whether early assessment of angiogenic markers may have utility in identifying high-risk pregnancies.

### Conclusions

Early changes in angiogenic proteins may have predictive utility in identifying women at risk of adverse birth outcomes in WLHIV receiving ART. While these findings need to be prospectively validated, early identification of women at increased risk of adverse birth outcomes may facilitate enhanced monitoring and referral to health care facilities equipped to manage high-risk pregnancies (Video).

## Figures and Tables

**Table S1:** Descriptive characteristics of the study population by ART treatment arm

Baseline Characteristic	Treatment Arm		*P* Value
	Efavirenz-Based ART, n=160	Lopinavir/ritonavir -Based ART, n=166	
Age (years)	30 [26, 33]	29.2 ± 5.3	0.623
BMI (kg/m^2^)	21.2 [19.6, 22.9]	21.6 [20.2, 23.2]	0.074
Socioeconomic status (tertile)			0.611
1	54 (36.2)	57 (35.6)	
2	68 (45.6)	67 (41.9)	
3	27 (18.1)	36 (22.)	
Gestational age enrolment (weeks)	23.6 [19.7, 27.6]	23.5 [19.4, 27.0]	0.711
Previous pregnancies			
0	13 (8.3)	7 (4.2)	0.275
1	15 (9.6)	20 (12.1)	
≥2	132 (82.2)	139 (83.6)	
Hemoglobin level (g/dL)	11.0 [10.2, 11.8]	11.1 [10.2, 11.7]	0.855
White blood cell count (cells/mm^3^)	4900 [4100, 6100]	5200 [4300, 6400]	0.134
Platelet count (x10^9^/L)	218.0 [177.5, 255.5]	201.0 [167.0, 239.5]	0.080
CD4+T-cell count (cells/mm^3^)	373.0 (269.3-497.8)	368.0 (280.5-507.5)	0.575
HIV RNA load (log_10_copies/mL)	4.286 [3.427, 4.837]	4.097 [3.328, 4.755]	0.468
**Outcome Characteristics**			
Gestational age delivery (weeks)	39 (37-40)	38 (37-39)	0.061
Birth weight (kg)	2910 (2680-3240)	2880 (2650-3210)	0.503
Preterm birth	24 (15.0)	31 (18.7)	0.376
Small-for-gestational age	44 (27.2)	37 (23.9)	0.502
Stillbirth	4 (2.5)	5 (3.0)	1.000
Placental malaria^1^	14 (10.2)	10 (7.1)	0.363

Continuous variables expressed as median (interquartile range), categorical variables expressed as n (%).

*Conroy et al. Angiogenic factors across pregnancy in women living with HIV. Am J Obstet Gynecol 2017*.

1 Placental malaria defined by a positive finding of a placental blood smear or PCR.

**Table S2:** Linear mixed effects models of angiogenic markers and preterm birth

	Ang-1		Ang-2		sFlt-1		PlGF		sEng	
	Beta	SE	Beta	SE	Beta	SE	Beta	SE	Beta	SE
**Fixed Terms**										
(Intercept)	2.13479	0.4445	2.17519	0.3960	−0.43520	0.5145	−3.26956	0.6119	2.13733	0.1760
Preterm	0.13387	0.1206	−0.15845	0.1068	0.17902	0.1499	−0.16196	0.1617	0.16745	0.0475
Gestational age (shifted)	−0.00844	0.0064	−0.06347	0.0053	0.15426	0.0094	0.36999	0.0260	0.04093	0.0030
Gestational age (shifted) squared							−0.01407	0.0010		
Maternal age	−0.00304	0.0120	−0.01471	0.0109	−0.00375	0.0132	0.00646	0.0162	−0.00102	0.0047
Enrolment BMI	0.00297	0.0161	0.00655	0.0144	−0.02036	0.0181	−0.00298	0.0216	−0.00455	0.0063
Gravidity	−0.02431	0.0277	0.01920	0.0253	−0.02479	0.0308	0.08150	0.0375	−0.01270	0.0109
Gestational age: treatment arm interaction	0.00132	0.0062	−0.00290	0.0063	0.00437	0.0058	−0.00402	0.0086	−0.00507	0.0025
Number of subjects	320		312		320		320		320	
Observations	1104		1049		1104		1104		1085	
LR Test against Null Model	χ^2^(1) = 1259, p > 0.05		χ^2^(1) = 2.233, p > 0.05		χ^2^(1) = 1.444, p > 0.05		χ^2^(1) = 1.020, p > 0.05		χ^2^(1) = 12.282, p < 0.0005*	

*Conroy et al. Angiogenic factors across pregnancy in women living with HIV. Am J Obstet Gynecol 2017*.

**Table S3:** Linear mixed effects models of angiogenic markers and small-for-gestational age

	Ang-1		Ang-2		sFlt-1		PlGF		sEng	
	Beta	SE	Beta	SE	Beta	SE	Beta	SE	Beta	SE
**Fixed Terms**										
(Intercept)	2.14191	0.4559	2.13246	0.4024	−0.39429	0.5229	−3.03817	0.6134	2.12674	0.1820
Stillbirth	−0.00712	0.1028	0.13317	0.0920	−0.03339	0.1140	−0.38094	0.1357	−0.00189	0.0408
Gestational age (shifted)	−0.00760	0.0064	−0.06247	0.0053	0.15138	0.0095	0.36286	0.0264	0.04084	0.0030
Gestational age (shifted) squared							−0.01379	0.0010		
Maternal age	−0.00374	0.0124	−0.01508	0.0111	−0.00339	0.0136	0.01062	0.0163	−0.00060	0.0049
Enrolment BMI	0.00301	0.0165	0.00489	0.0147	−0.01953	0.0185	−0.00981	0.0218	−0.00400	0.0066
Gravidity	−0.01963	0.0287	0.02506	0.0259	−0.02451	0.0319	0.06139	0.0379	−0.01134	0.0114
Gestational age: treatment arm interaction	0.00113	0.0063	−0.00296	0.0064	0.00519	0.0059	−0.00384	0.0087	−0.00445	0.0026
Number of subjects	311		303		311		311		311	
Observations	1080		1025		1080		1080		1061	
LR Test against Null Model	χ^2^(1) = 0.0, p > 0.05		χ^2^(1) = 2.128, p > 0.05		χ^2^(1) = 0.0878, p > 0.05		χ^2^(1) = 7.892, p < 0.005*		χ^2^(1) = 0.0025, p > 0.05	

*Conroy et al. Angiogenic factors across pregnancy in women living with HIV. Am J Obstet Gynecol 2017*.

**Table S4:** Linear mixed effects models of angiogenic markers and stillbirth

	Ang-1		Ang-2		sFlt-1		PlGF		sEng	
	Beta	SE	Beta	SE	Beta	SE	Beta	SE	Beta	SE
**Fixed Terms**										
(Intercept)	2.14007	0.4468	2.20069	0.3979	−0.40889	0.5146	−3.14340	0.5889	2.12762	0.1788
Stillbirth	0.01616	0.2756	−0.24706	0.2320	−0.34066	0.3784	−1.66380	0.3520	0.26459	0.1110
Gestational age (shifted)	−0.00887	0.0064	−0.06329	0.0053	0.15278	0.0093	0.36828	0.0259	0.04064	0.0030
Gestational age (shifted) squared							−0.01405	0.0010		
Maternal age	−0.00309	0.0120	−0.01486	0.0109	−0.00362	0.0132	0.00881	0.0156	−0.00154	0.0048
Enrolment BMI	0.00369	0.0161	0.00480	0.0145	−0.01959	0.0181	−0.00876	0.0208	−0.00297	0.0064
Gravidity	−0.02331	0.0278	0.01885	0.0253	−0.02359	0.0308	0.07530	0.0359	−0.01047	0.0111
Gestational age: treatment arm interaction	0.00149	0.0062	−0.00316	0.0063	0.00427	0.0058	−0.00421	0.0085	−0.00471	0.0026
Number of subjects	320		312		320		320		320	
Observations	1104		1049		1104		1104		1085	
LR Test against Null Model	χ^2^(1) = 0.0045, p > 0.05		χ^2^(1) = 1.129, p > 0.05		χ^2^(1) = 0.819, p > 0.05		χ^2^(1) = 21.595, p < 0.0001*		χ^2^(1) = 5.6717, p < 0.02*	

*Conroy et al. Angiogenic factors across pregnancy in women living with HIV. Am J Obstet Gynecol 2017*.

## References

[1] World Health Organization (2015). Global summary of the AIDS epidemic.

[2] Papp E., Mohammadi H., Loutfy M.R. (2015). HIV protease inhibitor use during pregnancy is associated with decreased progesterone levels, suggesting a potential mechanism contributing to fetal growth restriction. J Infect Dis.

[3] Chen J.Y., Ribaudo H.J., Souda S. (2012). Highly active antiretroviral therapy and adverse birth outcomes among HIV-infected women in Botswana. J Infect Dis.

[4] Machado E.S., Hofer C.B., Costa T.T. (2009). Pregnancy outcome in women infected with HIV-1 receiving combination antiretroviral therapy before versus after conception. Sex Transm Infect.

[5] Ekouevi D.K., Coffie P.A., Becquet R. (2008). Antiretroviral therapy in pregnant women with advanced HIV disease and pregnancy outcomes in Abidjan, Cote d'Ivoire. AIDS.

[6] Wimalasundera R.C., Larbalestier N., Smith J.H. (2002). Pre-eclampsia, antiretroviral therapy, and immune reconstitution. Lancet.

[7] Townsend C.L., Cortina-Borja M., Peckham C.S., Tookey P.A. (2007). Antiretroviral therapy and premature delivery in diagnosed HIV-infected women in the United Kingdom and Ireland. AIDS.

[8] Powis K.M., Kitch D., Ogwu A. (2011). Increased risk of preterm delivery among HIV-infected women randomized to protease versus nucleoside reverse transcriptase inhibitor-based HAART during pregnancy. J Infect Dis.

[9] Newell M.L., Bunders M.J. (2013). Safety of antiretroviral drugs in pregnancy and breastfeeding for mother and child. Curr Opin HIV AIDS.

[10] Hernandez S., Moren C., Lopez M. (2012). Perinatal outcomes, mitochondrial toxicity and apoptosis in HIV-treated pregnant women and in-utero-exposed newborn. AIDS.

[11] Zash R., Jacobson D.L., Diseko M. (2017). Comparative safety of antiretroviral treatment regimens in pregnancy. JAMA Pediatr.

[12] Nattabi B., Li J., Thompson S.C., Orach C.G., Earnest J. (2009). A systematic review of factors influencing fertility desires and intentions among people living with HIV/AIDS: implications for policy and service delivery. AIDS Behav.

[13] Kalk E., Schubert P., Bettinger J.A. (2017). Placental pathology in HIV infection at term: a comparison with HIV-uninfected women. Trop Med Int Health.

[14] Geva E., Ginzinger D.G., Zaloudek C.J., Moore D.H., Byrne A., Jaffe R.B. (2002). Human placental vascular development: vasculogenic and angiogenic (branching and nonbranching) transformation is regulated by vascular endothelial growth factor-a, angiopoietin-1, and angiopoietin-2. J Clin Endocrinol Metab.

[15] Charnock-Jones D.S., Kaufmann P., Mayhew T.M. (2004). Aspects of human fetoplacental vasculogenesis and angiogenesis, I: molecular regulation. Placenta.

[16] Levine R.J., Maynard S.E., Qian C. (2004). Circulating angiogenic factors and the risk of preeclampsia. N Engl J Med.

[17] Zhou Y., McMaster M., Woo K. (2002). Vascular endothelial growth factor ligands and receptors that regulate human cytotrophoblast survival are dysregulated in severe preeclampsia and hemolysis, elevated liver enzymes, and low platelets syndrome. Am J Pathol.

[18] Hirokoshi K., Maeshima Y., Kobayashi K. (2007). Elevated serum sFlt-1/Ang-2 ratio in women with preeclampsia. Nephron Clin Pract.

[19] Levine R.J., Lam C., Qian C. (2006). Soluble endoglin and other circulating antiangiogenic factors in preeclampsia. N Engl J Med.

[20] Govender N., Naicker T., Rajakumar A., Moodley J. (2013). Soluble fms-like tyrosine kinase-1 and soluble endoglin in HIV-associated preeclampsia. Eur J Obstet Gynecol Reprod Biol.

[21] Chaiworapongsa T., Romero R., Korzeniewski S.J. (2013). Maternal plasma concentrations of angiogenic/antiangiogenic factors in the third trimester of pregnancy to identify the patient at risk for stillbirth at or near term and severe late preeclampsia. Am J Obstet Gynecol.

[22] Romero R., Nien J.K., Espinoza J. (2008). A longitudinal study of angiogenic (placental growth factor) and anti-angiogenic (soluble endoglin and soluble vascular endothelial growth factor receptor-1) factors in normal pregnancy and patients destined to develop preeclampsia and deliver a small for gestational age neonate. J Matern Fetal Neonatal Med.

[23] Chaiworapongsa T., Romero R., Kusanovic J.P. (2010). Plasma soluble endoglin concentration in pre-eclampsia is associated with an increased impedance to flow in the maternal and fetal circulations. Ultrasound Obstet Gynecol.

[24] Easter S.R., Cantonwine D.E., Zera C.A., Lim K.-H., Parry S.I., McElrath T.F. (2016). Urinary tract infection during pregnancy, angiogenic factor profiles, and risk of preeclampsia. Am J Obstet Gynecol.

[25] Baltajian K., Bajracharya S., Salahuddin S. (2016). Sequential plasma angiogenic factors levels in women with suspected preeclampsia. Am J Obstet Gynecol.

[26] Yang J., Pearl M., DeLorenze G.N. (2016). Racial-ethnic differences in midtrimester maternal serum levels of angiogenic and antiangiogenic factors. Am J Obstet Gynecol.

[27] Holme A.M., Roland M.C., Henriksen T., Michelsen T.M. (2016). In vivo uteroplacental release of placental growth factor and soluble Fms-like tyrosine kinase-1 in normal and preeclamptic pregnancies. Am J Obstet Gynecol.

[28] Kim M.Y., Buyon J.P., Guerra M.M. (2016). Angiogenic factor imbalance early in pregnancy predicts adverse outcomes in patients with lupus and antiphospholipid antibodies: results of the PROMISSE study. Am J Obstet Gynecol.

[29] Wang Y., Tasevski V., Wallace E.M., Gallery E.D., Morris J.M. (2007). Reduced maternal serum concentrations of angiopoietin-2 in the first trimester precede intrauterine growth restriction associated with placental insufficiency. BJOG.

[30] Taylor R.N., Grimwood J., Taylor R.S., McMaster M.T., Fisher S.J., North R.A. (2003). Longitudinal serum concentrations of placental growth factor: evidence for abnormal placental angiogenesis in pathologic pregnancies. Am J Obstet Gynecol.

[31] Mijal R.S., Holzman C.B., Rana S., Karumanchi S.A., Wang J., Sikorskii A. (2012). Mid-pregnancy levels of angiogenic markers as indicators of pathways to preterm delivery. J Matern Fetal Neonatal Med.

[32] Romero R., Chaiworapongsa T., Erez O. (2010). An imbalance between angiogenic and anti-angiogenic factors precedes fetal death in a subset of patients: results of a longitudinal study. J Matern Fetal Neonatal Med.

[33] Andersen L.B., Dechend R., Karumanchi S.A. (2016). Early pregnancy angiogenic markers and spontaneous abortion: an Odense Child Cohort study. Am J Obstet Gynecol.

[34] Korzeniewski S.J., Romero R., Chaiworapongsa T. (2016). Maternal plasma angiogenic index-1 (placental growth factor/soluble vascular endothelial growth factor receptor-1) is a biomarker for the burden of placental lesions consistent with uteroplacental underperfusion: a longitudinal case-cohort study. Am J Obstet Gynecol.

[35] Redman C.W.G., Staff A.C. (2015). Preeclampsia, biomarkers, syncytiotrophoblast stress, and placental capacity. Am J Obstet Gynecol.

[36] Fisher S.J. (2015). Why is placentation abnormal in preeclampsia?. Am J Obstet Gynecol.

[37] Yancopoulos G.D., Davis S., Gale N.W., Rudge J.S., Wiegand S.J., Holash J. (2000). Vascular-specific growth factors and blood vessel formation. Nature.

[38] Krauss T., Pauer H.U., Augustin H.G. (2004). Prospective analysis of placenta growth factor (PlGF) concentrations in the plasma of women with normal pregnancy and pregnancies complicated by preeclampsia. Hypertens Pregnancy.

[39] Kendall R.L., Thomas K.A. (1993). Inhibition of vascular endothelial cell growth factor activity by an endogenously encoded soluble receptor. Proc Natl Acad Sci U S A.

[40] Gregory A.L., Xu G., Sotov V., Letarte M. (2014). Review: the enigmatic role of endoglin in the placenta. Placenta.

[41] Liu Z., Lebrin F., Maring J.A. (2014). Endoglin is dispensable for vasculogenesis, but required for vascular endothelial growth factor-induced angiogenesis. PLoS One.

[42] Natureeba P., Ades V., Luwedde F. (2014). Lopinavir/ritonavir-based antiretroviral treatment (ART) versus efavirenz-based ART for the prevention of malaria among HIV-infected pregnant women. J Infect Dis.

[43] Young S., Murray K., Mwesigwa J. (2012). Maternal nutritional status predicts adverse birth outcomes among HIV-infected rural Ugandan women receiving combination antiretroviral therapy. PLoS One.

[44] Villar J., Cheikh Ismail L., Victora C.G. (2014). International standards for newborn weight, length, and head circumference by gestational age and sex: the Newborn Cross-Sectional Study of the Intergrowth-21st Project. Lancet.

[45] R Core Team. R: A Language and Environment for Statistical Computing. R Foundation for Statistical Computing, Vienna, Austria. 2017. http://www.R-project.org/.

[46] Bates D. (2015). Fitting linear mixed-effects models using lme4. J Stat Software.

[47] United Nations Children's Fund and World Health Organization. Low birthweight: country, regional and global estimates. UNICEF New York: 2004.

[48] Chaiworapongsa T., Romero R., Tarca A. (2009). A subset of patients destined to develop spontaneous preterm labor has an abnormal angiogenic/anti-angiogenic profile in maternal plasma: evidence in support of pathophysiologic heterogeneity of preterm labor derived from a longitudinal study. J Matern Fetal Neonatal Med.

[49] McDonald C.R., Darling A.M., Conroy A.L. (2015). Inflammatory and angiogenic factors at mid-pregnancy are associated with spontaneous preterm birth in a cohort of Tanzanian women. PLoS One.

[50] Darling A.M., McDonald C.R., Conroy A.L. (2014). Angiogenic and inflammatory biomarkers in midpregnancy and small-for-gestational-age outcomes in Tanzania. Am J Obstet Gynecol.

[51] Joffe G.M., Esterlitz J.R., Levine R.J. (1998). The relationship between abnormal glucose tolerance and hypertensive disorders of pregnancy in healthy nulliparous women. Calcium for Preeclampsia Prevention (CPEP) study group. Am J Obstet Gynecol.

[52] Bolin M., Wiberg-Itzel E., Wikström A.-K. (2009). Angiopoietin-1/angiopoietin-2 ratio for prediction of preeclampsia. Am J Hypertens.

[53] Govender N., Naicker T., Moodley J. (2013). Maternal imbalance between pro-angiogenic and anti-angiogenic factors in HIV-infected women with pre-eclampsia. Cardiovasc J Afr.

[54] Graham S.M., Rajwans N., Jaoko W. (2013). Endothelial activation biomarkers increase after HIV-1 acquisition: plasma vascular cell adhesion molecule-1 predicts disease progression. AIDS.

[55] Graham S.M., Rajwans N., Tapia K.A. (2013). A prospective study of endothelial activation biomarkers, including plasma angiopoietin-1 and angiopoietin-2, in Kenyan women initiating antiretroviral therapy. BMC Infect Dis.

[56] Urbinati C., Nicoli S., Giacca M. (2009). HIV-1 Tat and heparan sulfate proteoglycan interaction: a novel mechanism of lymphocyte adhesion and migration across the endothelium. Blood.

[57] Das J.R., Gutkind J.S., Ray P.E. (2016). Circulating fibroblast growth factor-2, HIV-Tat, and vascular endothelial cell growth factor-A in HIV-infected children with renal disease activate Rho-A and Src in cultured renal endothelial cells. PLoS One.

[58] Plunkett B.A., Fitchev P., Doll J.A. (2008). Decreased expression of pigment epithelium derived factor (PEDF), an inhibitor of angiogenesis, in placentas of unexplained stillbirths. Reprod Biol.

[59] Gavard J.A. (2017). Gestational weight gain and maternal and neonatal outcomes in underweight pregnant women: a population-based historical cohort study. Matern Child Health J.

[60] Koss C.A., Natureeba P., Plenty A. (2014). Risk factors for preterm birth among HIV-infected pregnant Ugandan women randomized to lopinavir/ritonavir- or efavirenz-based antiretroviral therapy. J Acquir Immune Defic Syndr (1999).

