# Supplementary material for: Altered angiogenesis as a common mechanism underlying preterm birth, small for gestational age, and stillbirth in women living with HIV
Source: Am J Obstet Gynecol. 2017 Dec;217(6):684.e1–684.e17. doi: 10.1016/j.ajog.2017.10.003 (PMC5723571; doi:10.1016/j.ajog.2017.10.003)
Supplement: Supplementary Data [file mmc2.pptx]

## Slide 1
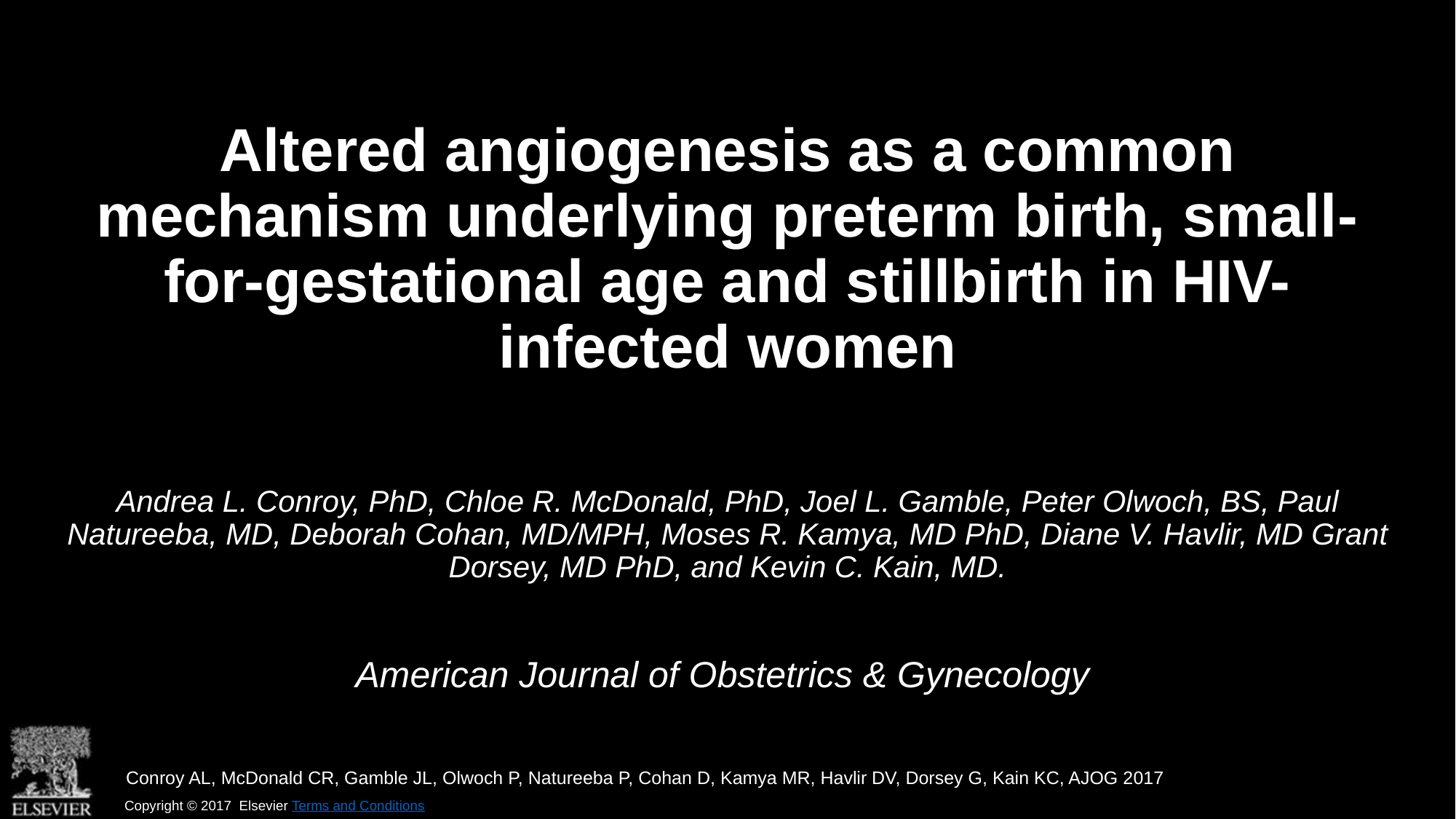

# Altered angiogenesis as a common mechanism underlying preterm birth, small-for-gestational age and stillbirth in HIV-infected women Andrea L. Conroy, PhD, Chloe R. McDonald, PhD, Joel L. Gamble, Peter Olwoch, BS, Paul Natureeba, MD, Deborah Cohan, MD/MPH, Moses R. Kamya, MD PhD, Diane V. Havlir, MD Grant Dorsey, MD PhD, and Kevin C. Kain, MD.   American Journal of Obstetrics & Gynecology
Conroy AL, McDonald CR, Gamble JL, Olwoch P, Natureeba P, Cohan D, Kamya MR, Havlir DV, Dorsey G, Kain KC, AJOG 2017
Copyright © 2017 Elsevier Terms and Conditions

## Slide 2
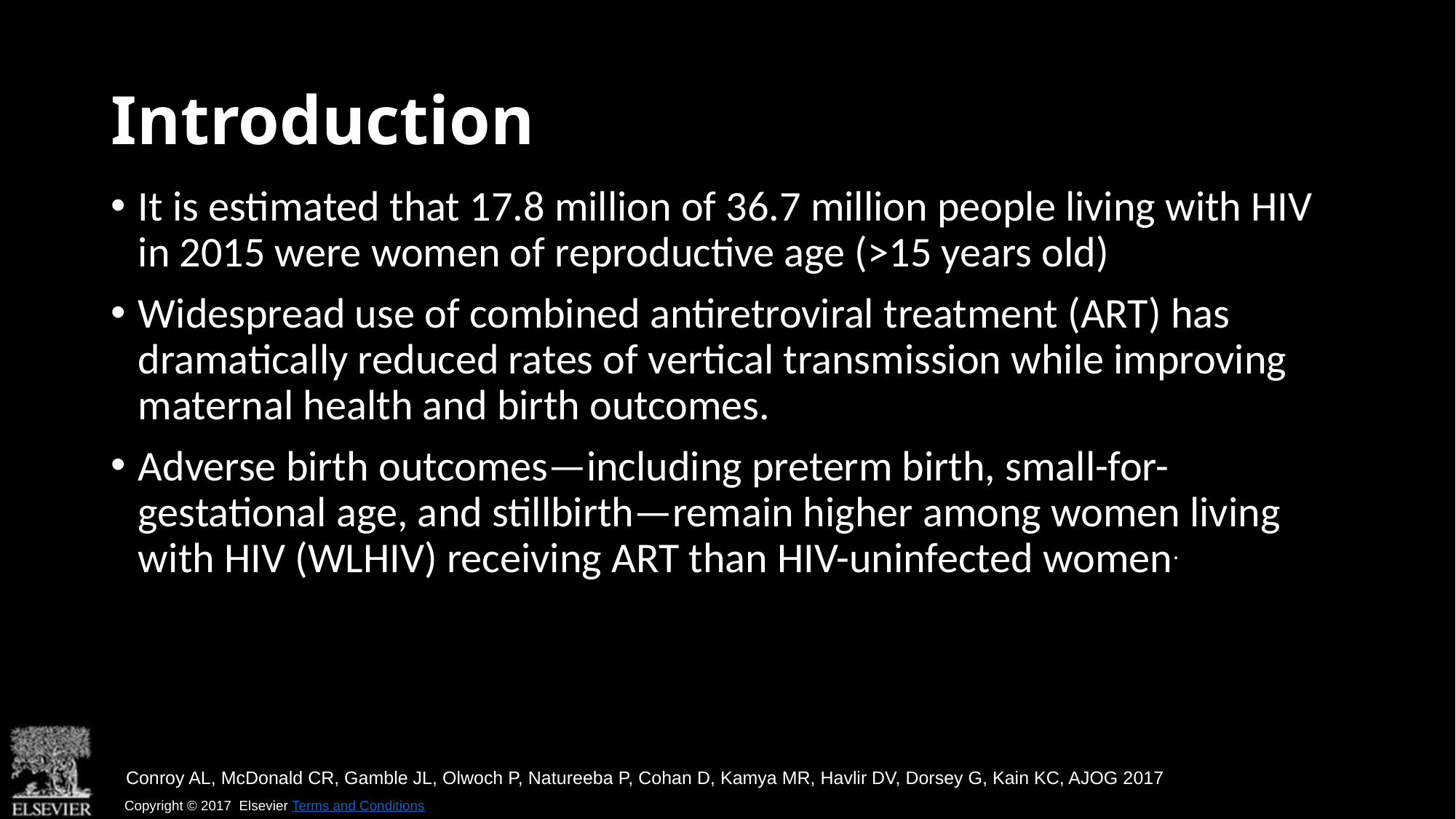

# Introduction
It is estimated that 17.8 million of 36.7 million people living with HIV in 2015 were women of reproductive age (>15 years old)
Widespread use of combined antiretroviral treatment (ART) has dramatically reduced rates of vertical transmission while improving maternal health and birth outcomes.
Adverse birth outcomes—including preterm birth, small-for-gestational age, and stillbirth—remain higher among women living with HIV (WLHIV) receiving ART than HIV-uninfected women.
Conroy AL, McDonald CR, Gamble JL, Olwoch P, Natureeba P, Cohan D, Kamya MR, Havlir DV, Dorsey G, Kain KC, AJOG 2017
Copyright © 2017 Elsevier Terms and Conditions

## Slide 3
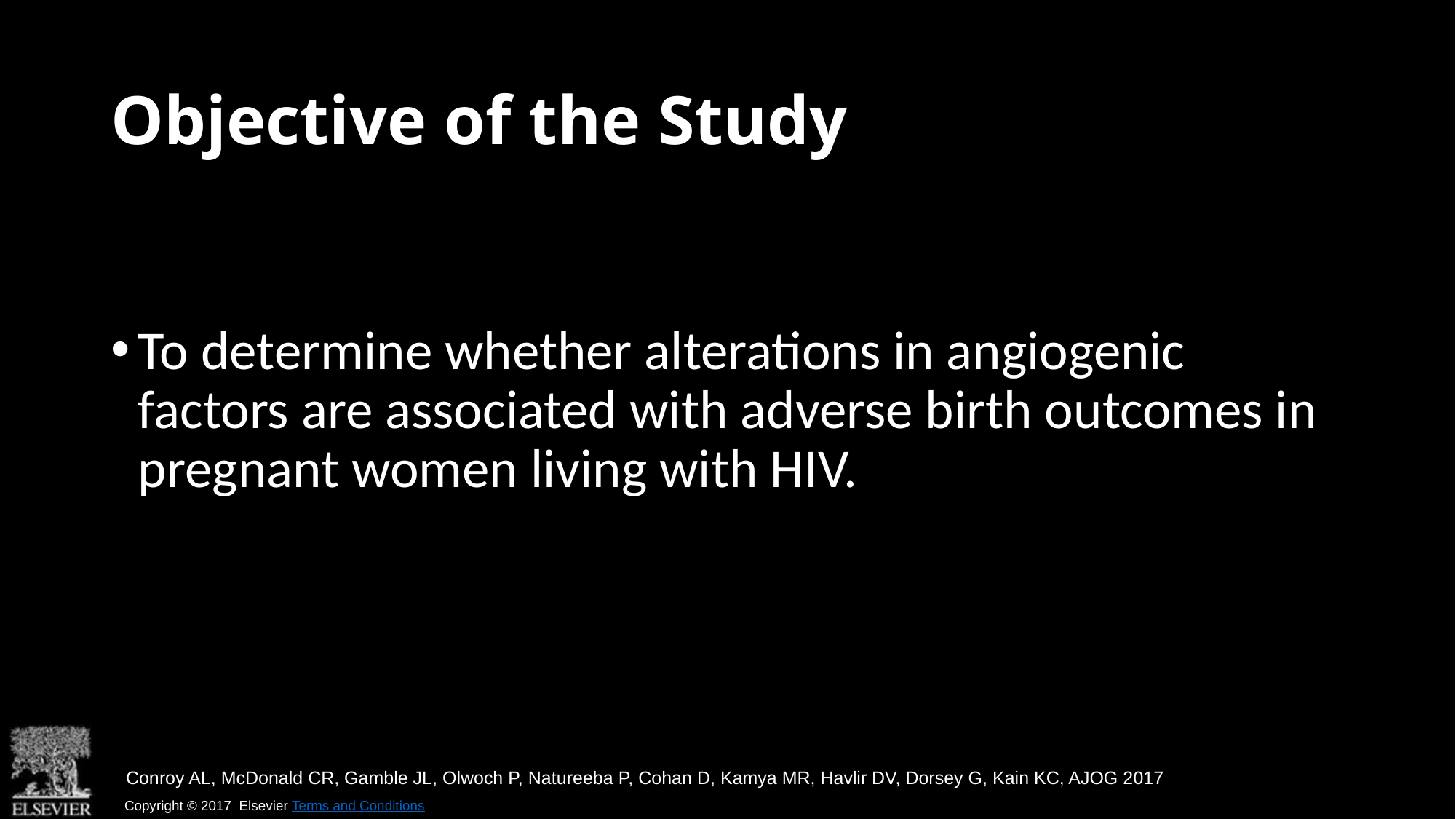

# Objective of the Study
To determine whether alterations in angiogenic factors are associated with adverse birth outcomes in pregnant women living with HIV.
Conroy AL, McDonald CR, Gamble JL, Olwoch P, Natureeba P, Cohan D, Kamya MR, Havlir DV, Dorsey G, Kain KC, AJOG 2017
Copyright © 2017 Elsevier Terms and Conditions

## Slide 4
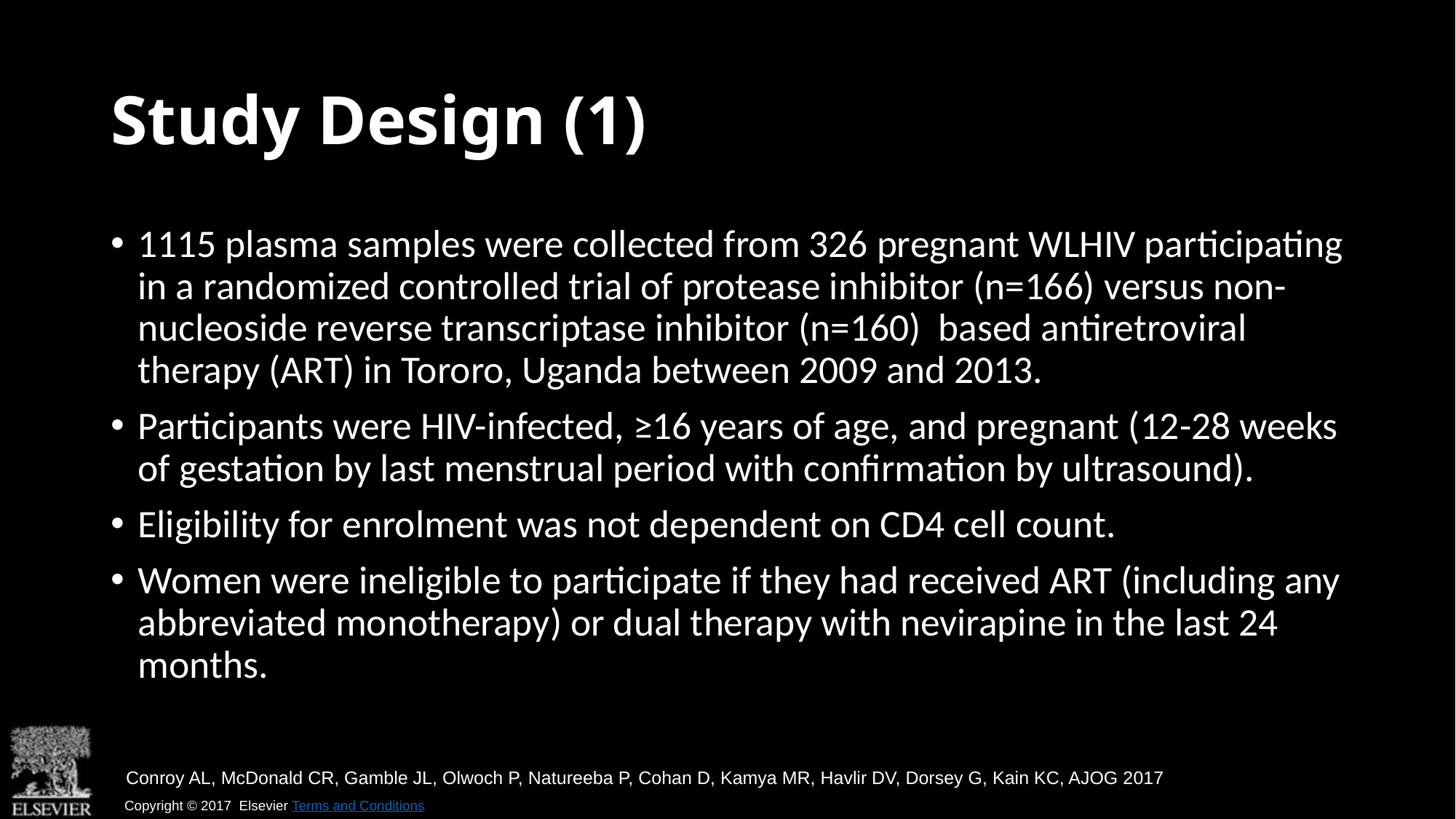

# Study Design (1)
1115 plasma samples were collected from 326 pregnant WLHIV participating in a randomized controlled trial of protease inhibitor (n=166) versus non-nucleoside reverse transcriptase inhibitor (n=160) based antiretroviral therapy (ART) in Tororo, Uganda between 2009 and 2013.
Participants were HIV-infected, ≥16 years of age, and pregnant (12-28 weeks of gestation by last menstrual period with confirmation by ultrasound).
Eligibility for enrolment was not dependent on CD4 cell count.
Women were ineligible to participate if they had received ART (including any abbreviated monotherapy) or dual therapy with nevirapine in the last 24 months.
Conroy AL, McDonald CR, Gamble JL, Olwoch P, Natureeba P, Cohan D, Kamya MR, Havlir DV, Dorsey G, Kain KC, AJOG 2017
Copyright © 2017 Elsevier Terms and Conditions

## Slide 5
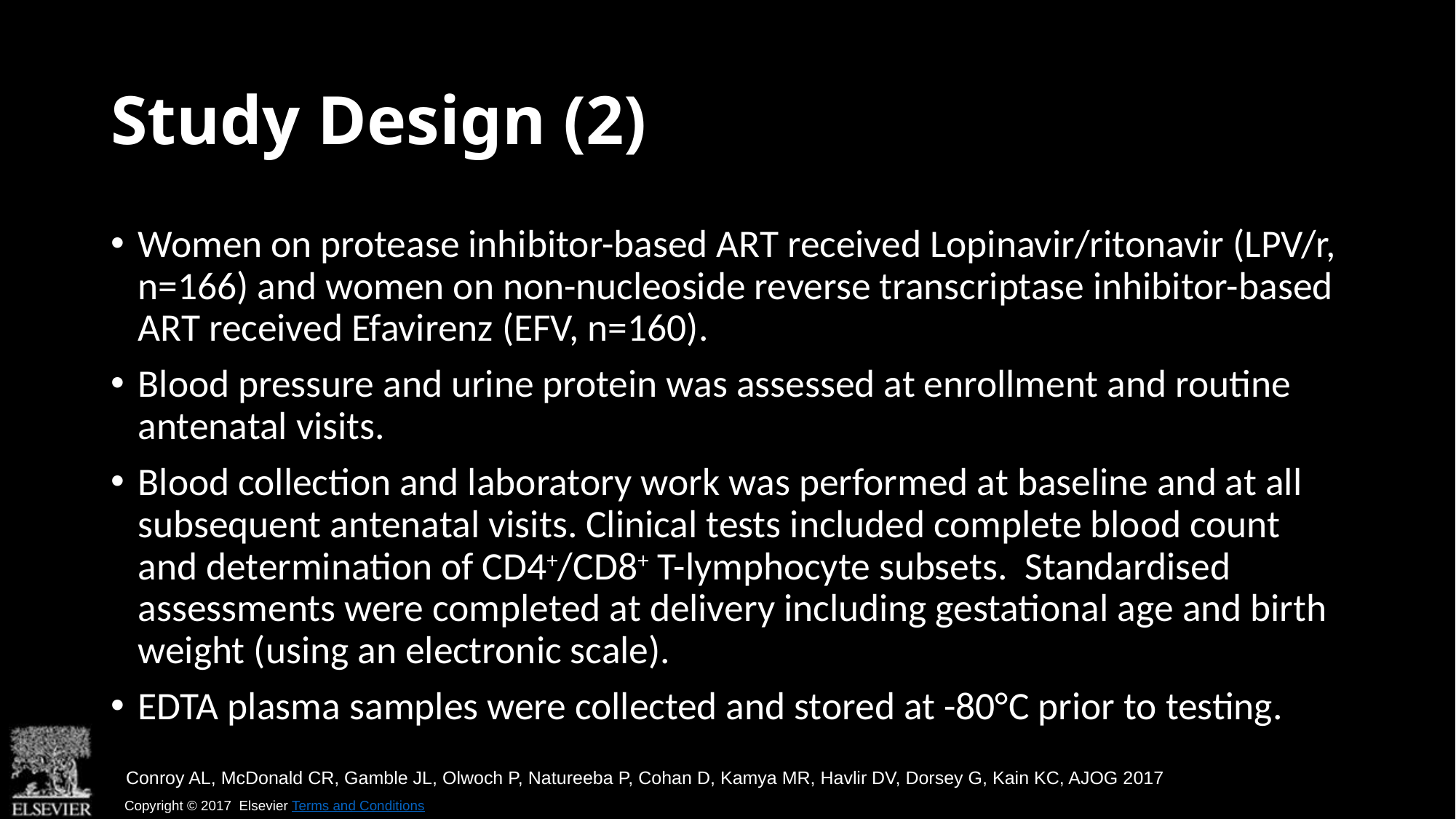

# Study Design (2)
Women on protease inhibitor-based ART received Lopinavir/ritonavir (LPV/r, n=166) and women on non-nucleoside reverse transcriptase inhibitor-based ART received Efavirenz (EFV, n=160).
Blood pressure and urine protein was assessed at enrollment and routine antenatal visits.
Blood collection and laboratory work was performed at baseline and at all subsequent antenatal visits. Clinical tests included complete blood count and determination of CD4+/CD8+ T-lymphocyte subsets. Standardised assessments were completed at delivery including gestational age and birth weight (using an electronic scale).
EDTA plasma samples were collected and stored at -80°C prior to testing.
Conroy AL, McDonald CR, Gamble JL, Olwoch P, Natureeba P, Cohan D, Kamya MR, Havlir DV, Dorsey G, Kain KC, AJOG 2017
Copyright © 2017 Elsevier Terms and Conditions

## Slide 6
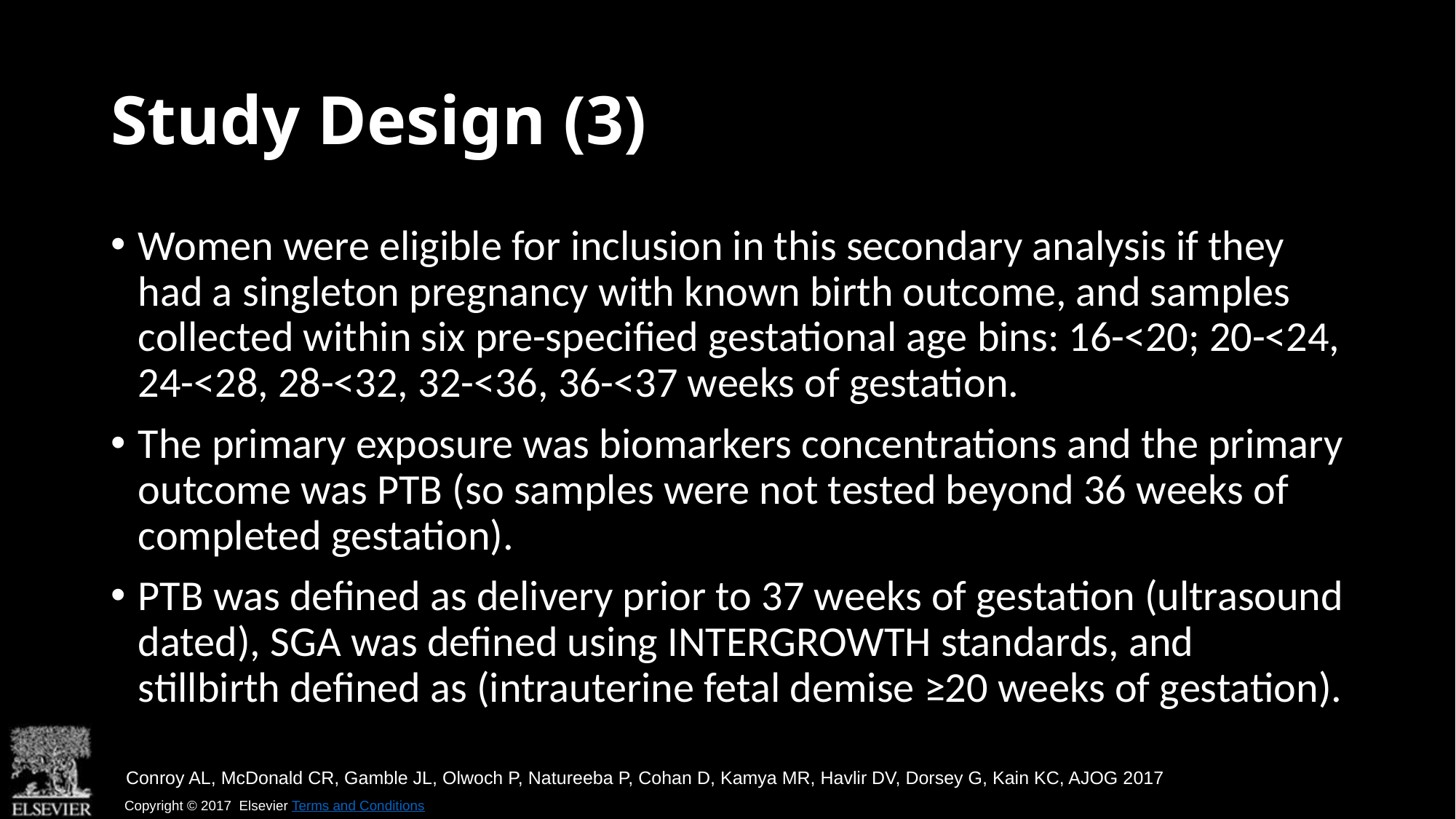

# Study Design (3)
Women were eligible for inclusion in this secondary analysis if they had a singleton pregnancy with known birth outcome, and samples collected within six pre-specified gestational age bins: 16-<20; 20-<24, 24-<28, 28-<32, 32-<36, 36-<37 weeks of gestation.
The primary exposure was biomarkers concentrations and the primary outcome was PTB (so samples were not tested beyond 36 weeks of completed gestation).
PTB was defined as delivery prior to 37 weeks of gestation (ultrasound dated), SGA was defined using INTERGROWTH standards, and stillbirth defined as (intrauterine fetal demise ≥20 weeks of gestation).
Conroy AL, McDonald CR, Gamble JL, Olwoch P, Natureeba P, Cohan D, Kamya MR, Havlir DV, Dorsey G, Kain KC, AJOG 2017
Copyright © 2017 Elsevier Terms and Conditions

## Slide 7
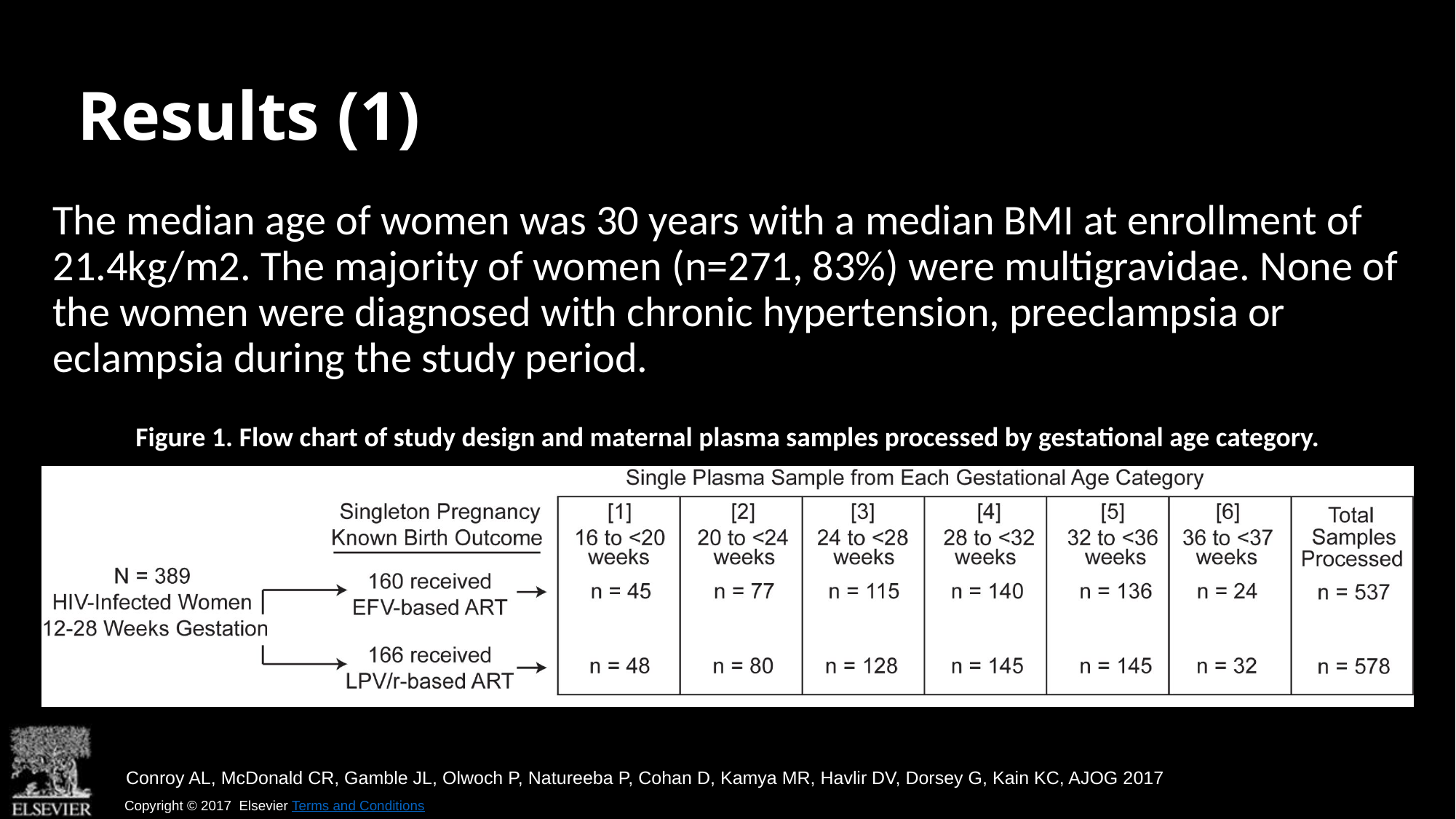

# Results (1)
The median age of women was 30 years with a median BMI at enrollment of 21.4kg/m2. The majority of women (n=271, 83%) were multigravidae. None of the women were diagnosed with chronic hypertension, preeclampsia or eclampsia during the study period.
Figure 1. Flow chart of study design and maternal plasma samples processed by gestational age category.
Conroy AL, McDonald CR, Gamble JL, Olwoch P, Natureeba P, Cohan D, Kamya MR, Havlir DV, Dorsey G, Kain KC, AJOG 2017
Copyright © 2017 Elsevier Terms and Conditions

## Slide 8
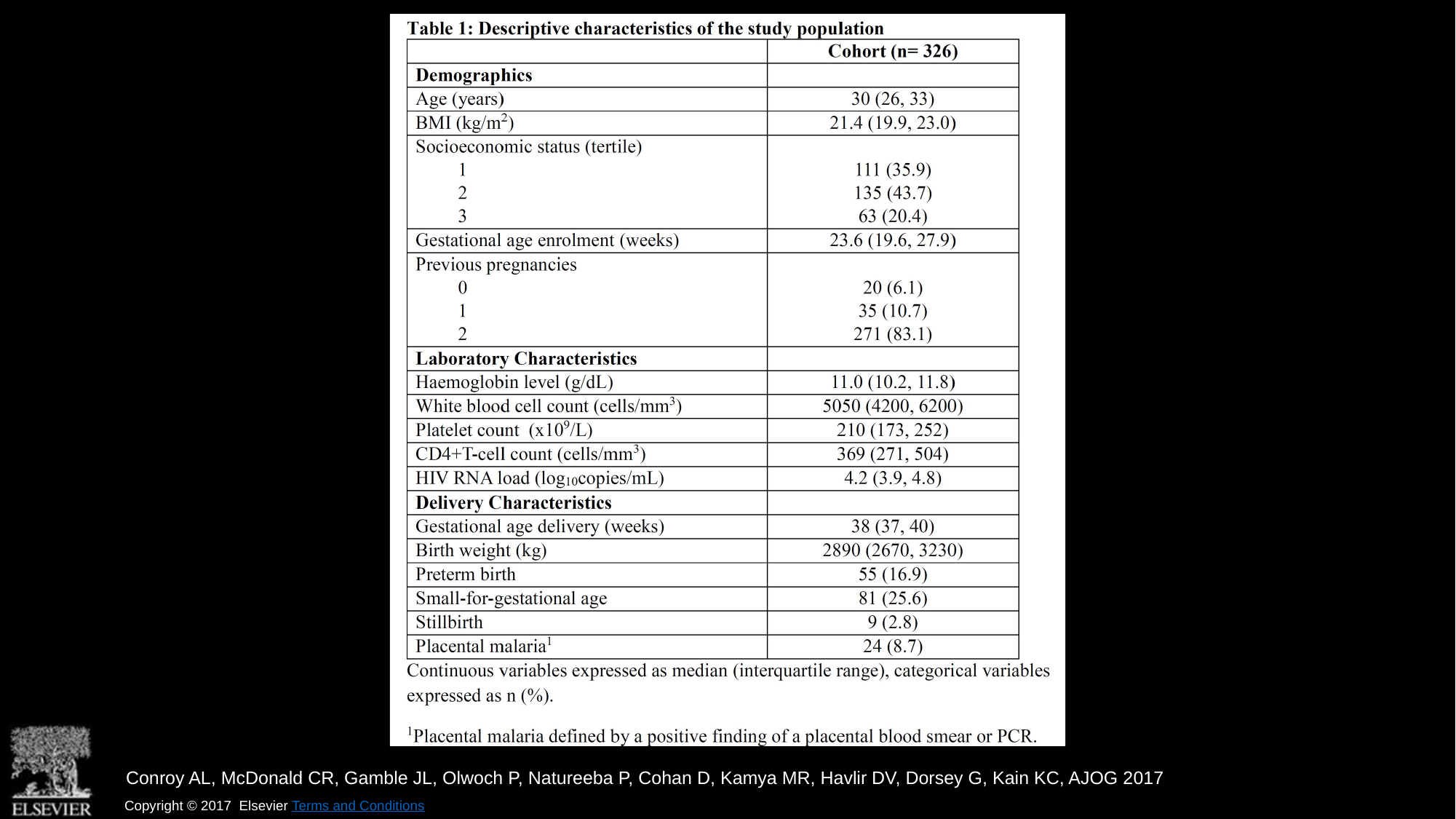

Conroy AL, McDonald CR, Gamble JL, Olwoch P, Natureeba P, Cohan D, Kamya MR, Havlir DV, Dorsey G, Kain KC, AJOG 2017
Copyright © 2017 Elsevier Terms and Conditions

## Slide 9
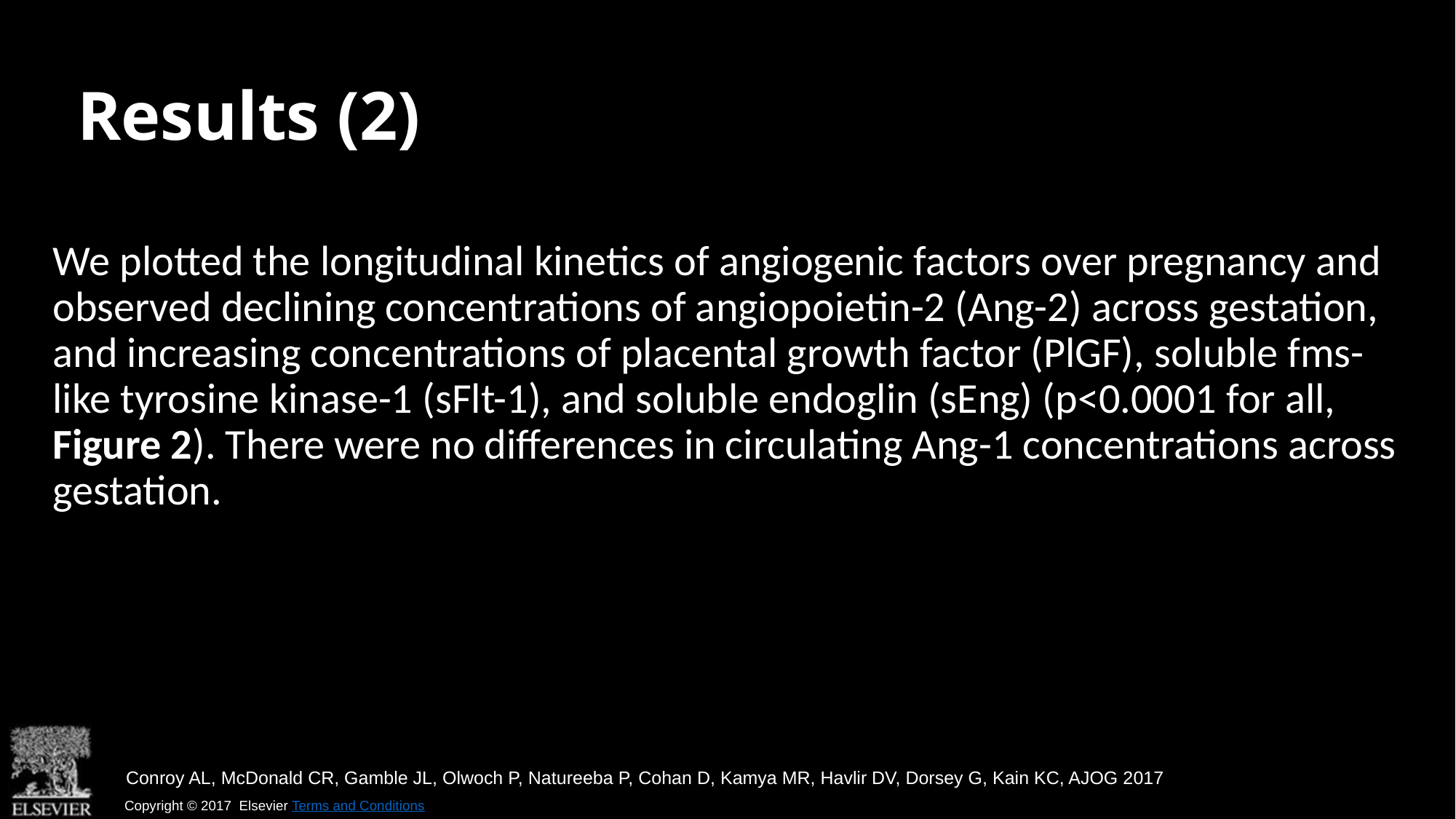

# Results (2)
We plotted the longitudinal kinetics of angiogenic factors over pregnancy and observed declining concentrations of angiopoietin-2 (Ang-2) across gestation, and increasing concentrations of placental growth factor (PlGF), soluble fms-like tyrosine kinase-1 (sFlt-1), and soluble endoglin (sEng) (p<0.0001 for all, Figure 2). There were no differences in circulating Ang-1 concentrations across gestation.
Conroy AL, McDonald CR, Gamble JL, Olwoch P, Natureeba P, Cohan D, Kamya MR, Havlir DV, Dorsey G, Kain KC, AJOG 2017
Copyright © 2017 Elsevier Terms and Conditions

## Slide 10
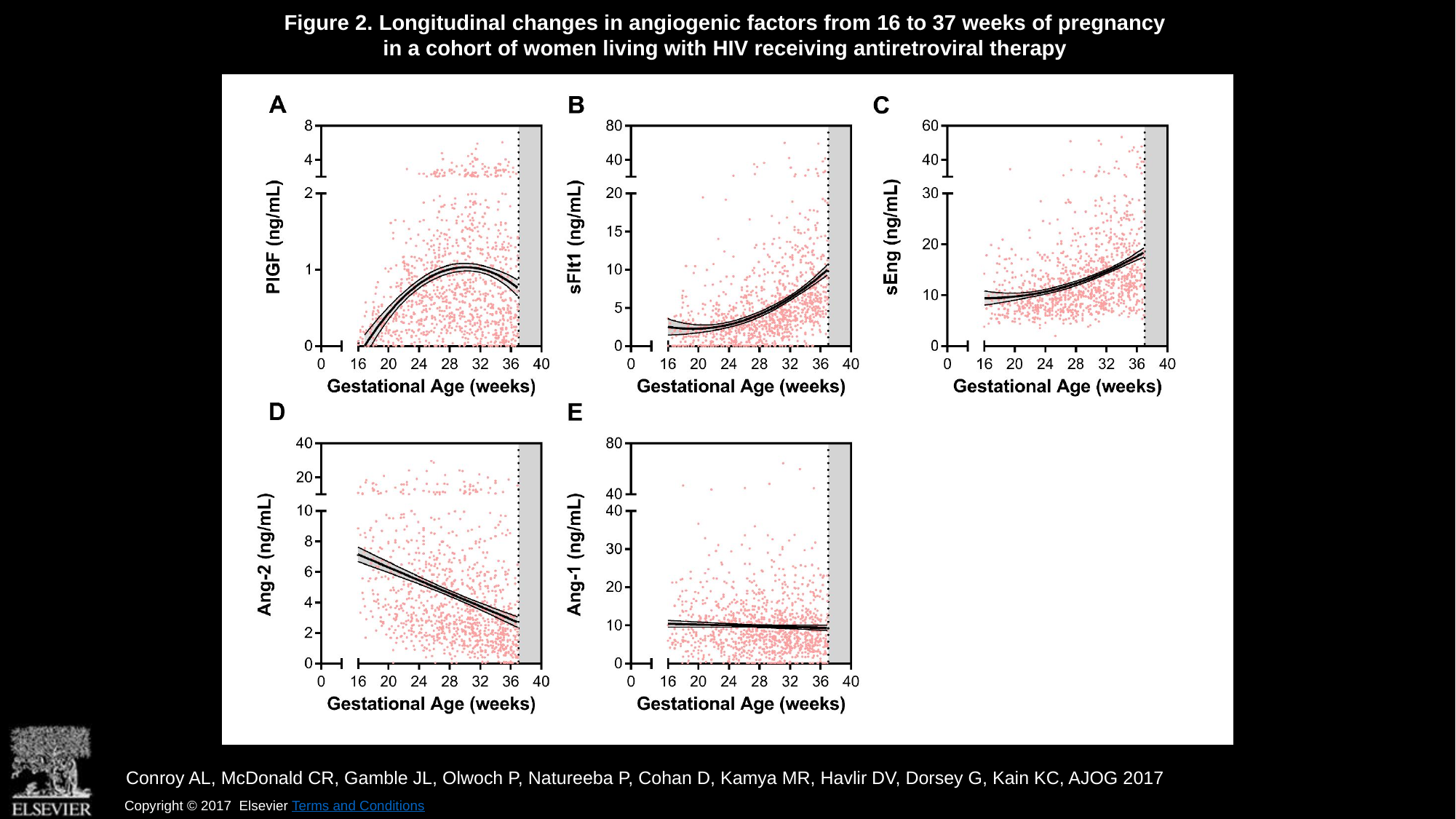

Figure 2. Longitudinal changes in angiogenic factors from 16 to 37 weeks of pregnancy
in a cohort of women living with HIV receiving antiretroviral therapy
Conroy AL, McDonald CR, Gamble JL, Olwoch P, Natureeba P, Cohan D, Kamya MR, Havlir DV, Dorsey G, Kain KC, AJOG 2017
Copyright © 2017 Elsevier Terms and Conditions

## Slide 11
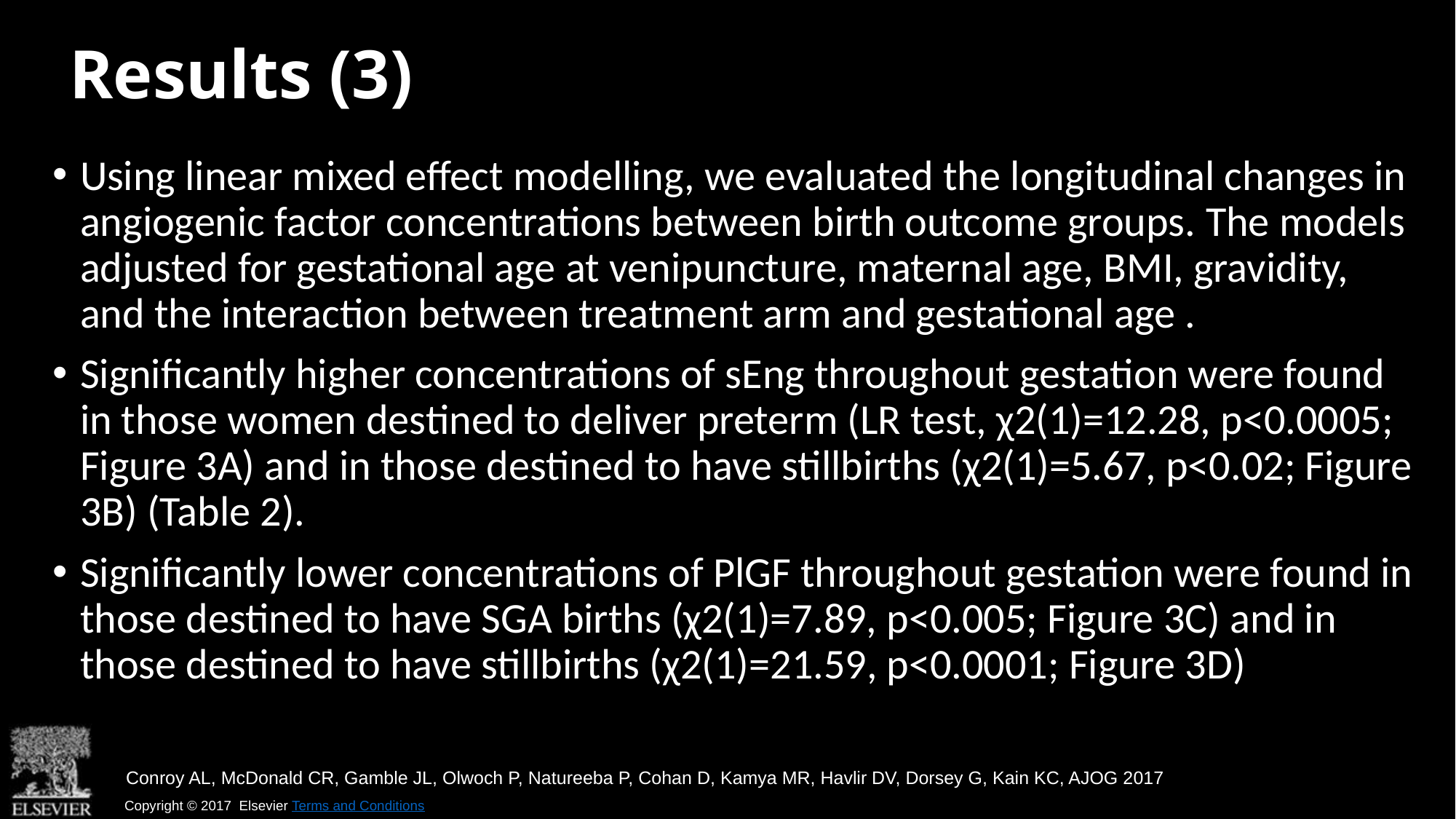

# Results (3)
Using linear mixed effect modelling, we evaluated the longitudinal changes in angiogenic factor concentrations between birth outcome groups. The models adjusted for gestational age at venipuncture, maternal age, BMI, gravidity, and the interaction between treatment arm and gestational age .
Significantly higher concentrations of sEng throughout gestation were found in those women destined to deliver preterm (LR test, χ2(1)=12.28, p<0.0005; Figure 3A) and in those destined to have stillbirths (χ2(1)=5.67, p<0.02; Figure 3B) (Table 2).
Significantly lower concentrations of PlGF throughout gestation were found in those destined to have SGA births (χ2(1)=7.89, p<0.005; Figure 3C) and in those destined to have stillbirths (χ2(1)=21.59, p<0.0001; Figure 3D)
Conroy AL, McDonald CR, Gamble JL, Olwoch P, Natureeba P, Cohan D, Kamya MR, Havlir DV, Dorsey G, Kain KC, AJOG 2017
Copyright © 2017 Elsevier Terms and Conditions

## Slide 12
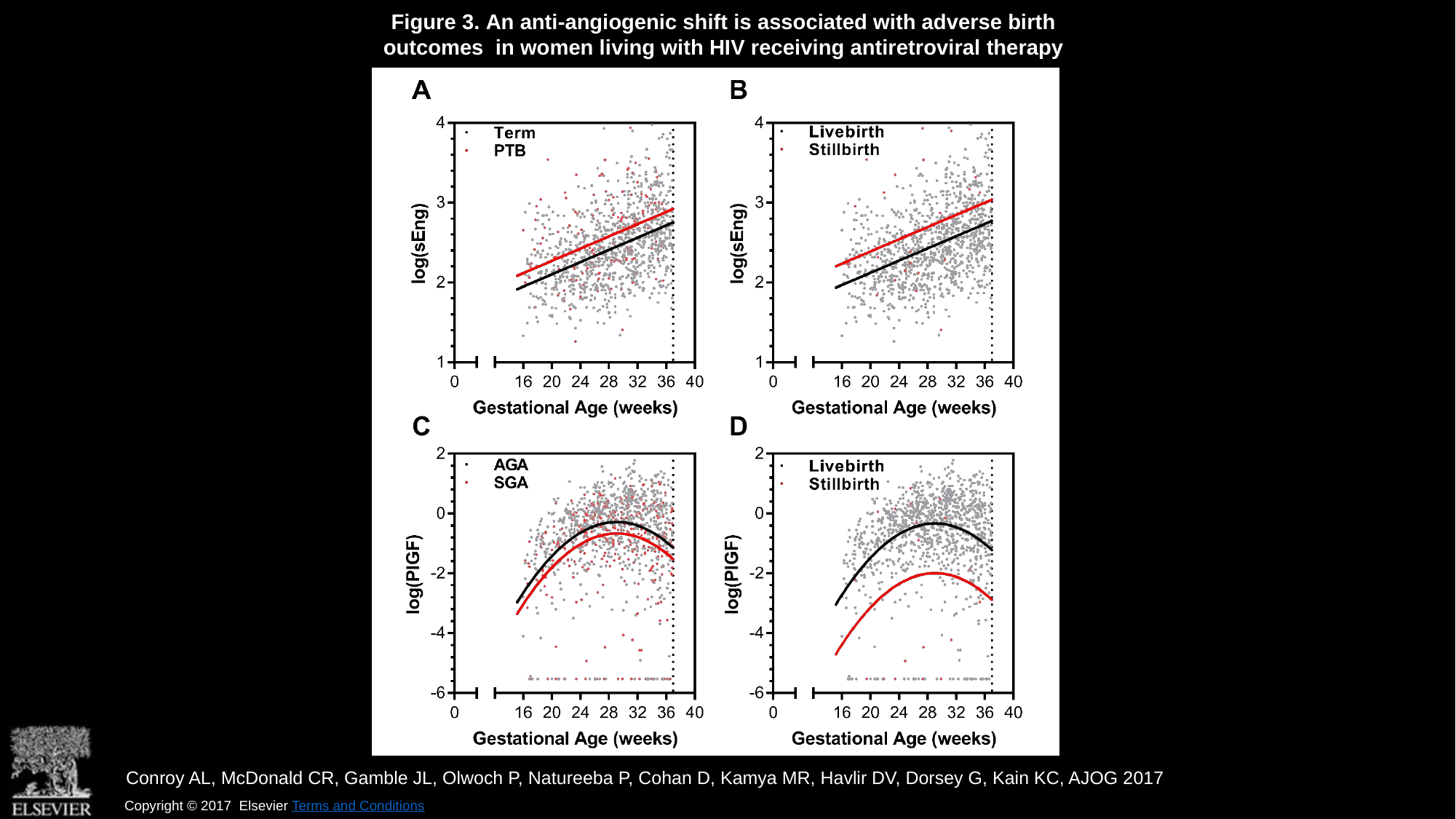

Figure 3. An anti-angiogenic shift is associated with adverse birth
outcomes in women living with HIV receiving antiretroviral therapy
Conroy AL, McDonald CR, Gamble JL, Olwoch P, Natureeba P, Cohan D, Kamya MR, Havlir DV, Dorsey G, Kain KC, AJOG 2017
Copyright © 2017 Elsevier Terms and Conditions

## Slide 13
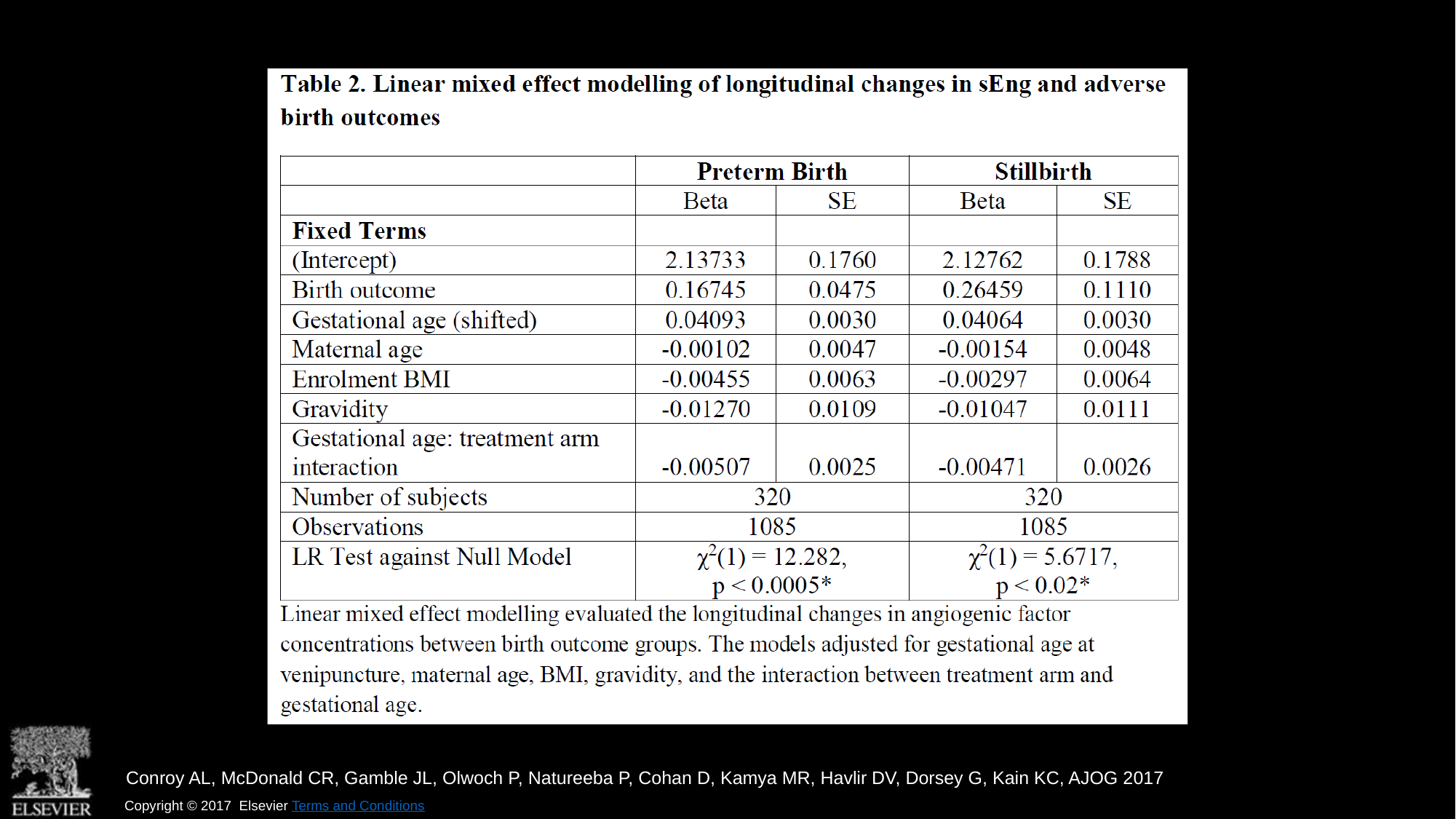

Conroy AL, McDonald CR, Gamble JL, Olwoch P, Natureeba P, Cohan D, Kamya MR, Havlir DV, Dorsey G, Kain KC, AJOG 2017
Copyright © 2017 Elsevier Terms and Conditions

## Slide 14
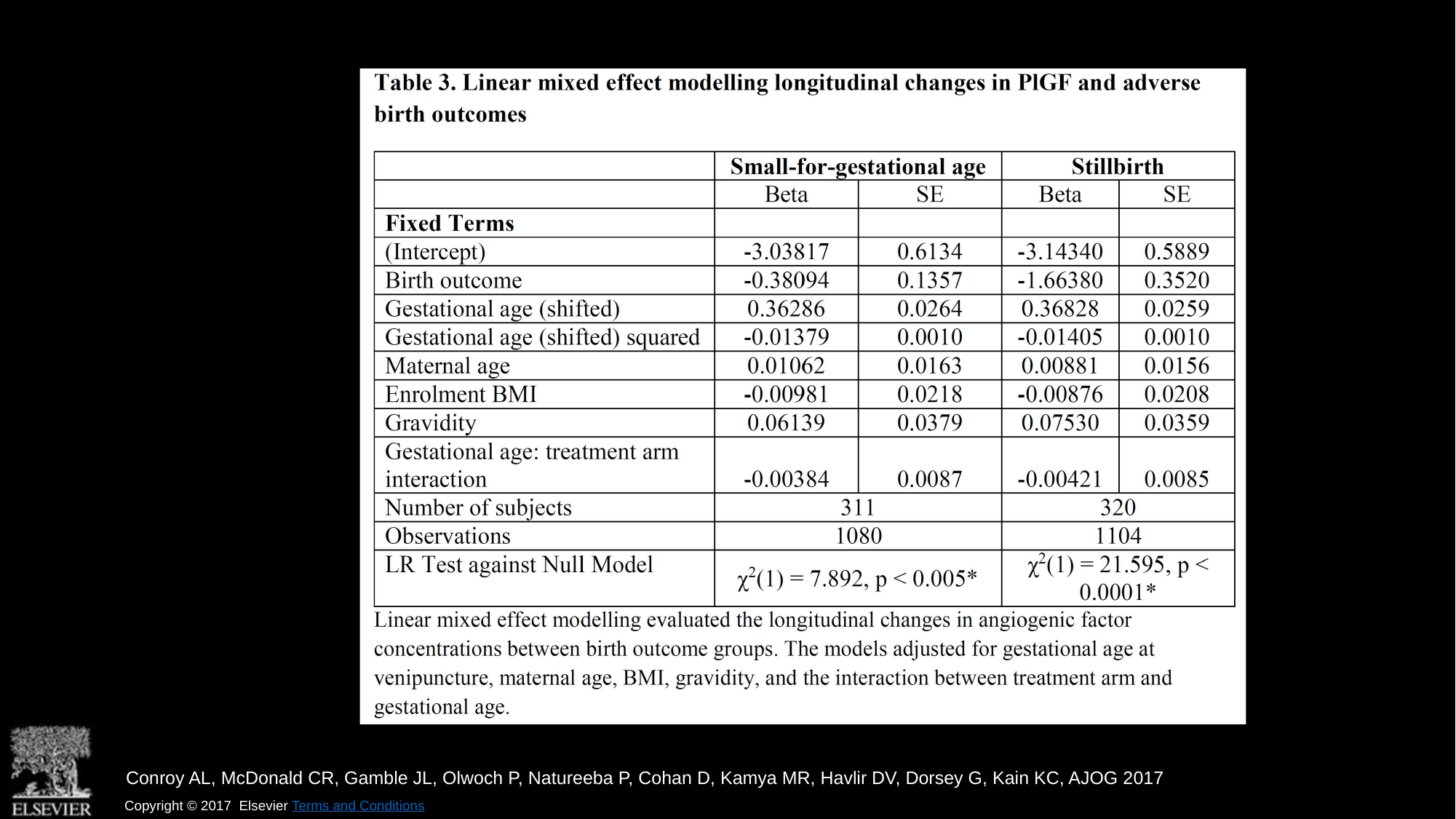

Conroy AL, McDonald CR, Gamble JL, Olwoch P, Natureeba P, Cohan D, Kamya MR, Havlir DV, Dorsey G, Kain KC, AJOG 2017
Copyright © 2017 Elsevier Terms and Conditions

## Slide 15
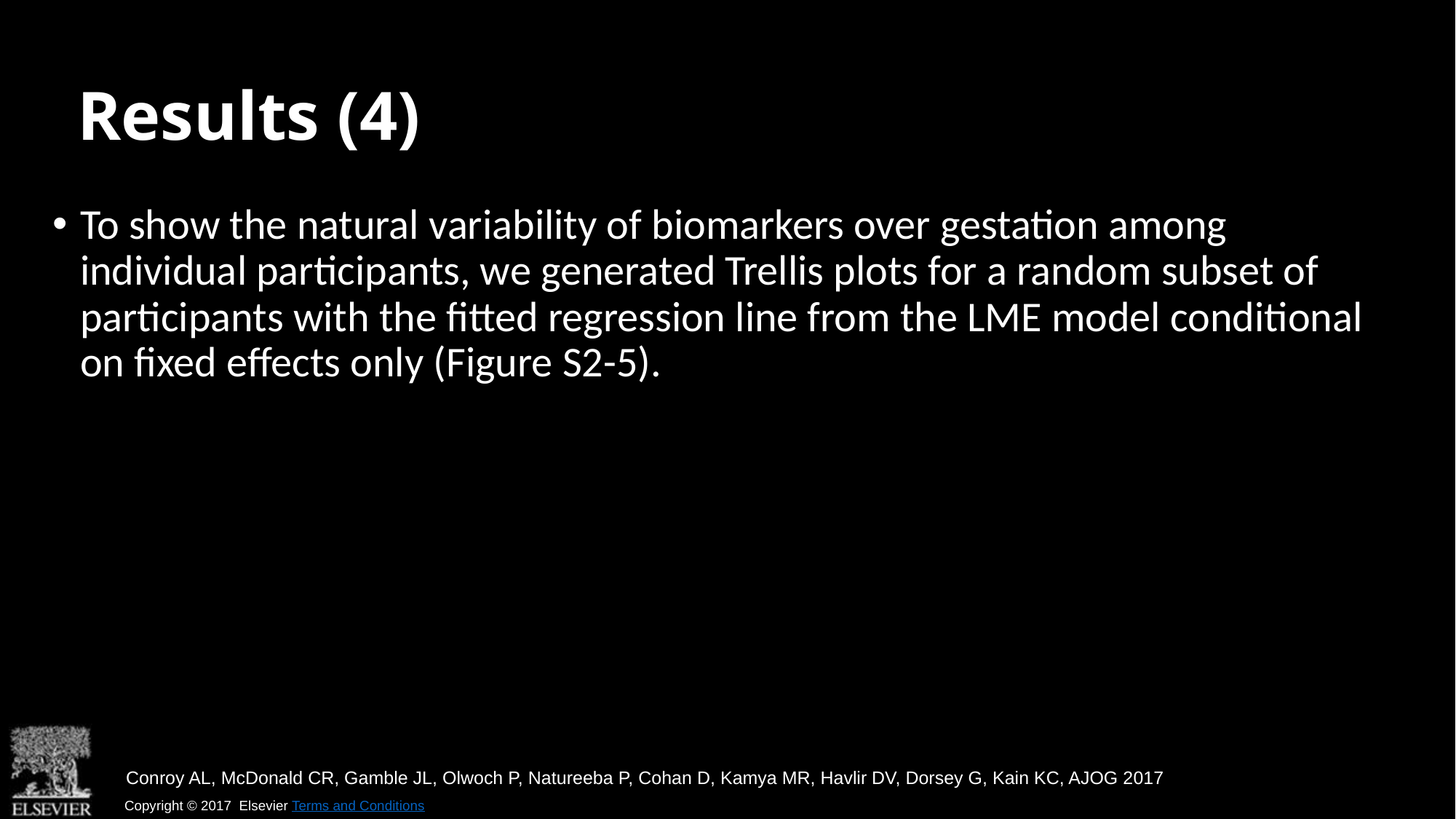

# Results (4)
To show the natural variability of biomarkers over gestation among individual participants, we generated Trellis plots for a random subset of participants with the fitted regression line from the LME model conditional on fixed effects only (Figure S2-5).
Conroy AL, McDonald CR, Gamble JL, Olwoch P, Natureeba P, Cohan D, Kamya MR, Havlir DV, Dorsey G, Kain KC, AJOG 2017
Copyright © 2017 Elsevier Terms and Conditions

## Slide 16
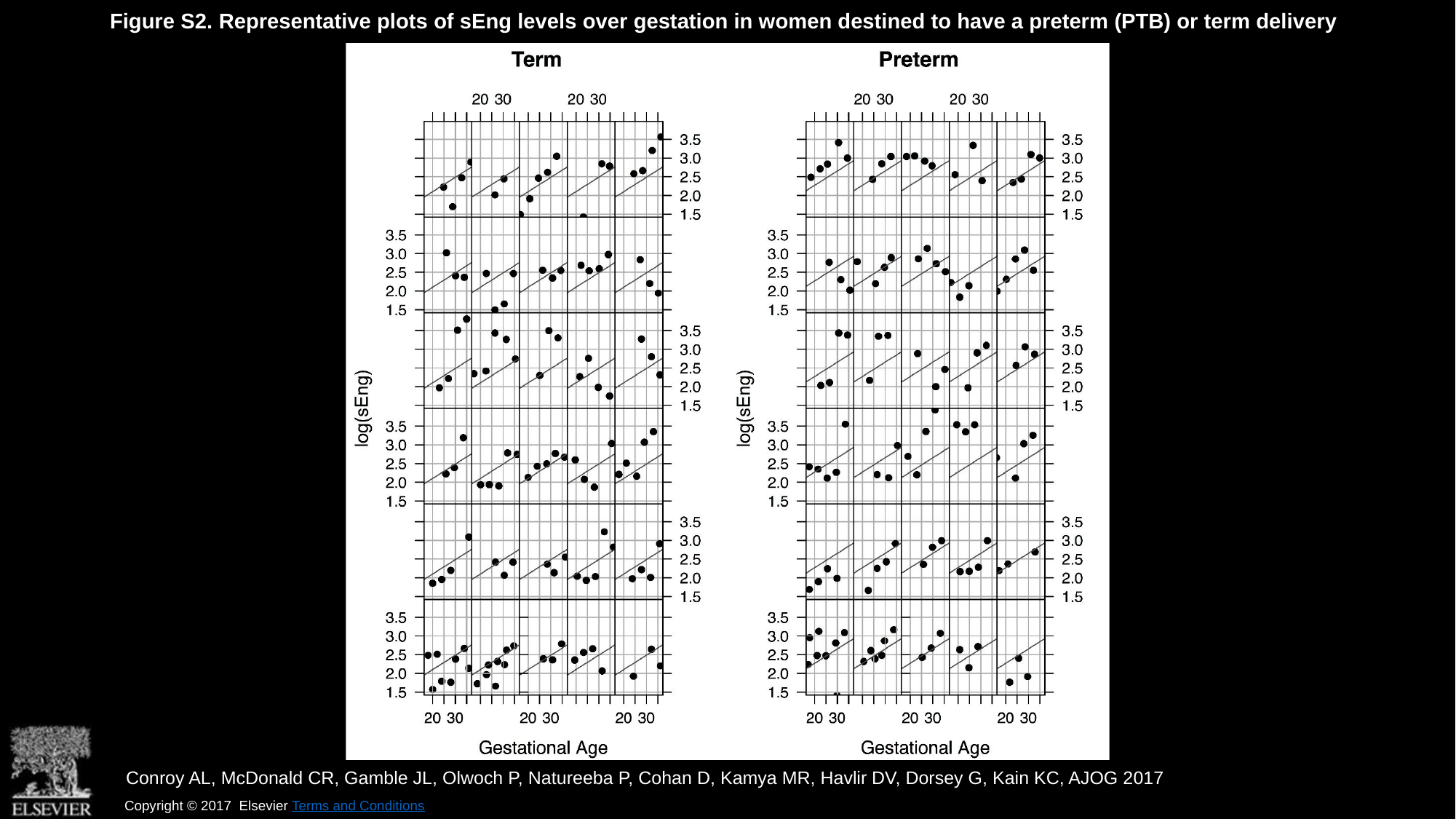

Figure S2. Representative plots of sEng levels over gestation in women destined to have a preterm (PTB) or term delivery
Conroy AL, McDonald CR, Gamble JL, Olwoch P, Natureeba P, Cohan D, Kamya MR, Havlir DV, Dorsey G, Kain KC, AJOG 2017
Copyright © 2017 Elsevier Terms and Conditions

## Slide 17
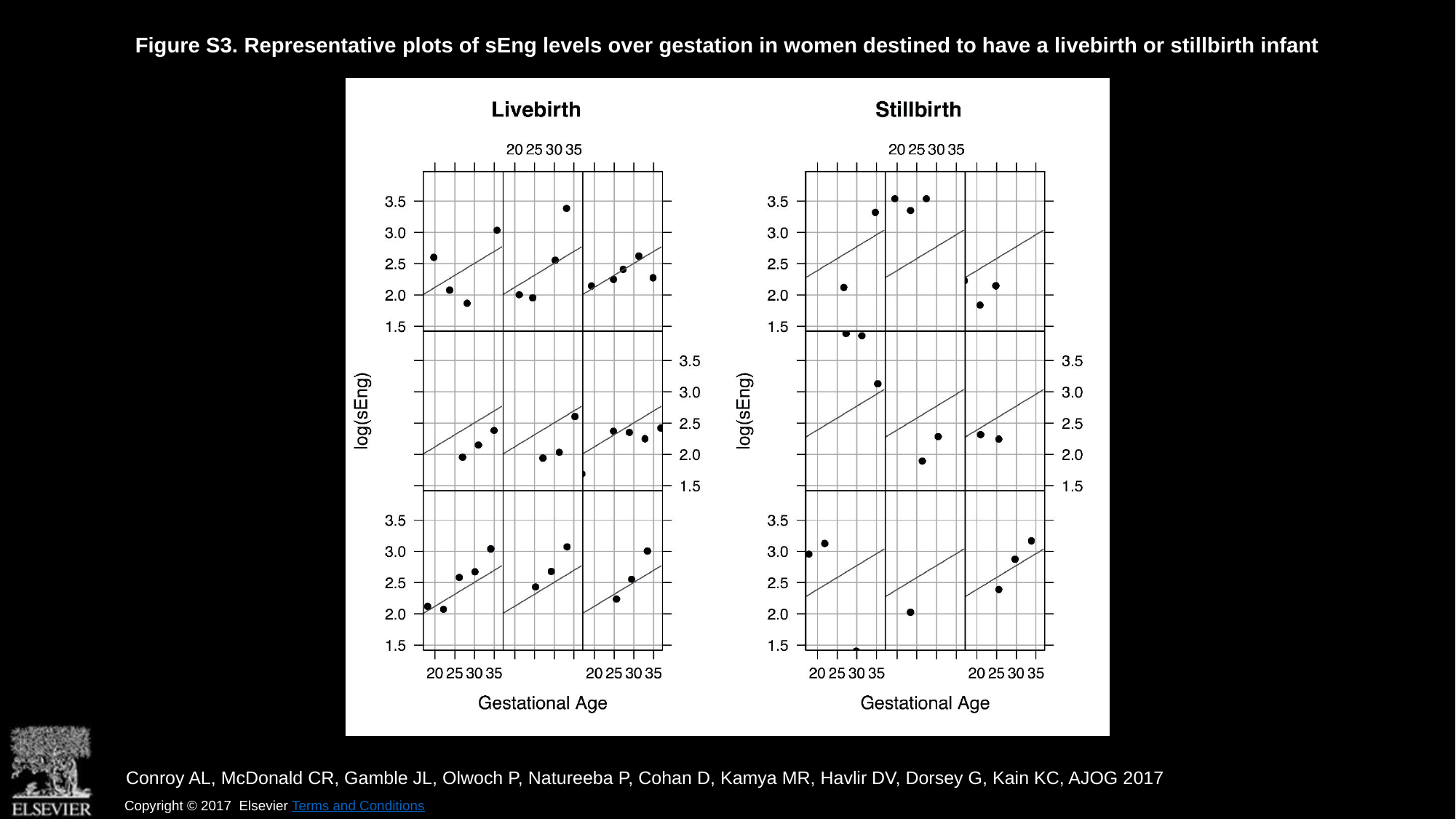

Figure S3. Representative plots of sEng levels over gestation in women destined to have a livebirth or stillbirth infant
Conroy AL, McDonald CR, Gamble JL, Olwoch P, Natureeba P, Cohan D, Kamya MR, Havlir DV, Dorsey G, Kain KC, AJOG 2017
Copyright © 2017 Elsevier Terms and Conditions

## Slide 18
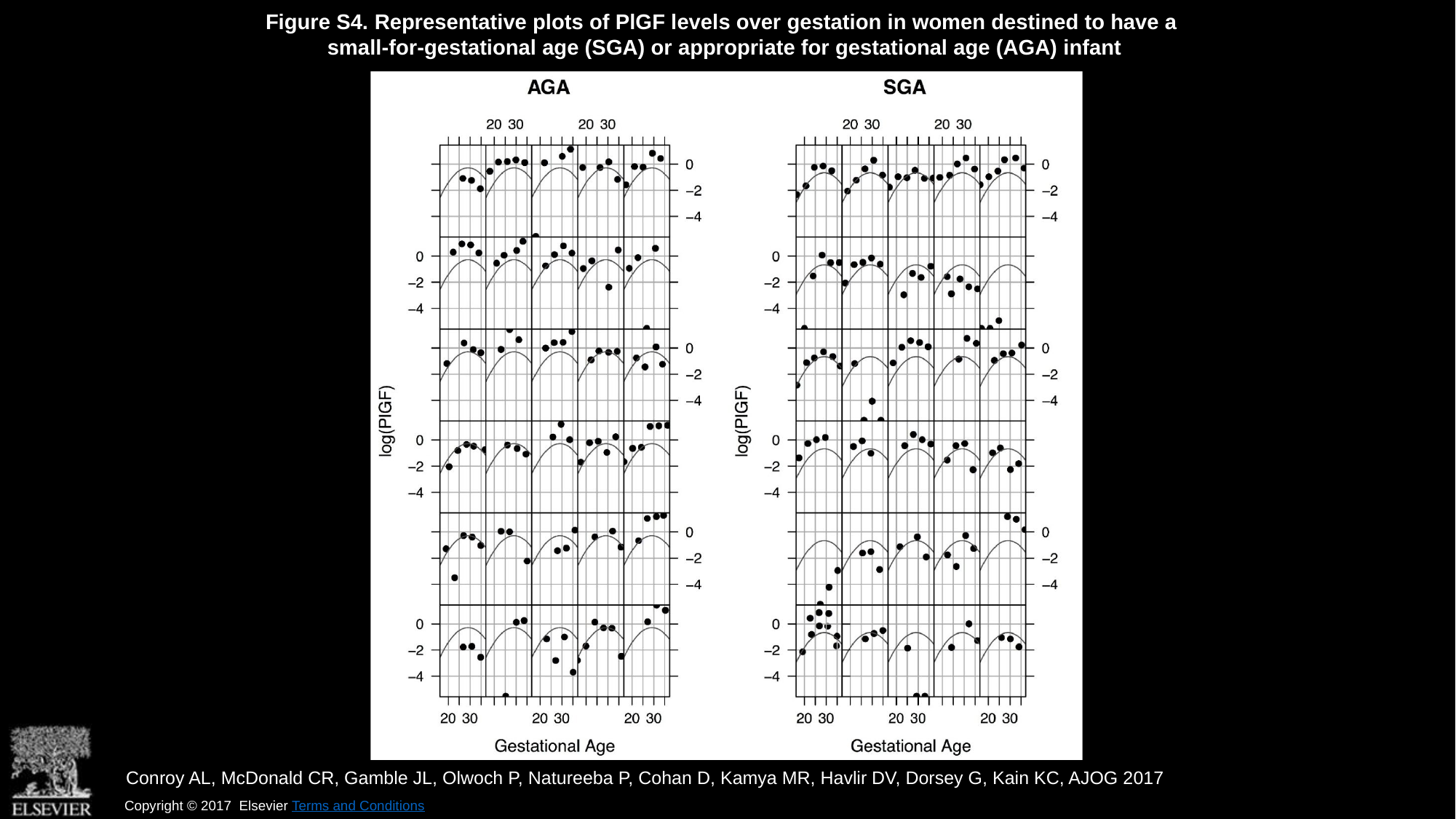

Figure S4. Representative plots of PlGF levels over gestation in women destined to have a
small-for-gestational age (SGA) or appropriate for gestational age (AGA) infant
Conroy AL, McDonald CR, Gamble JL, Olwoch P, Natureeba P, Cohan D, Kamya MR, Havlir DV, Dorsey G, Kain KC, AJOG 2017
Copyright © 2017 Elsevier Terms and Conditions

## Slide 19
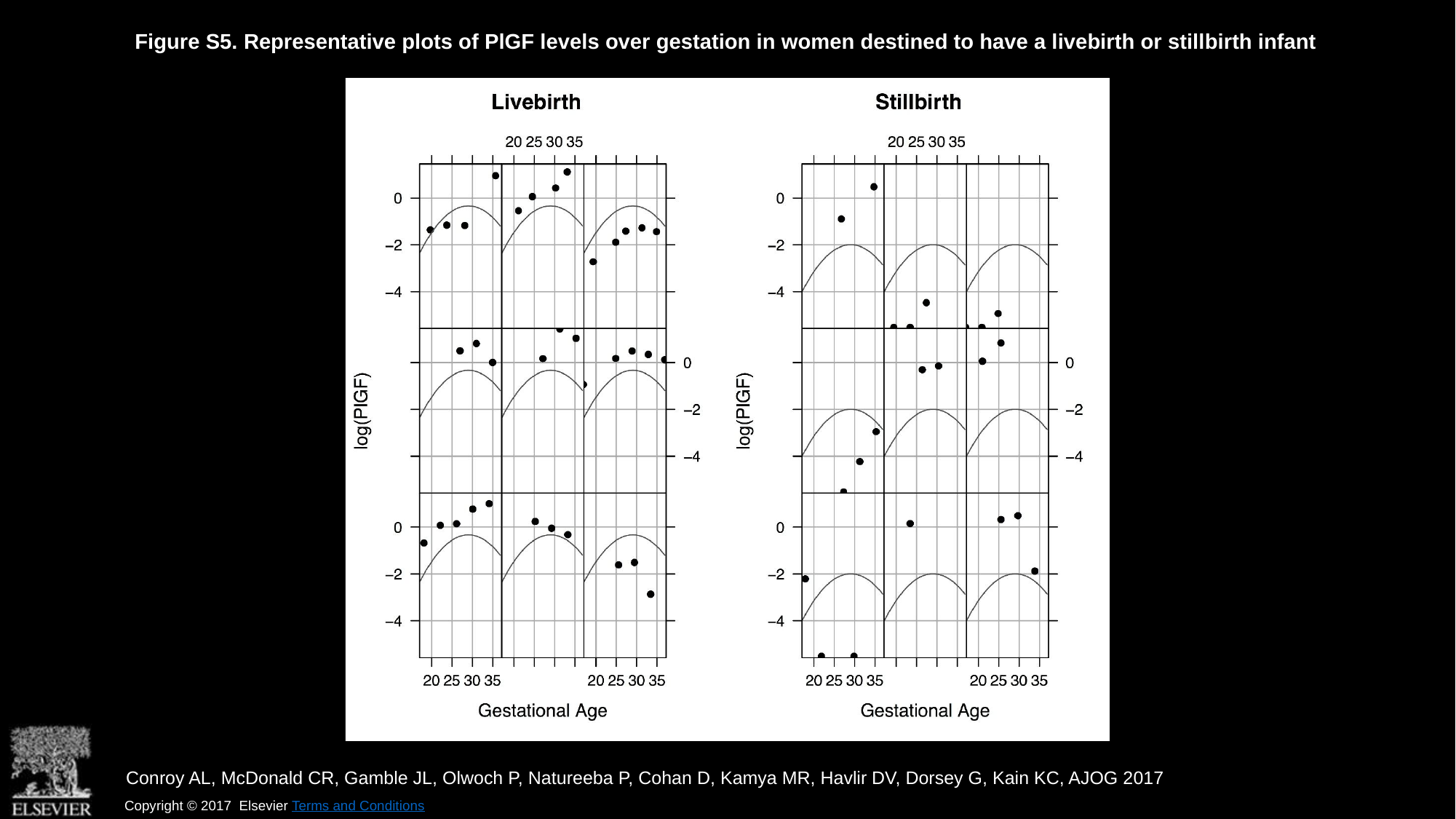

Figure S5. Representative plots of PlGF levels over gestation in women destined to have a livebirth or stillbirth infant
Conroy AL, McDonald CR, Gamble JL, Olwoch P, Natureeba P, Cohan D, Kamya MR, Havlir DV, Dorsey G, Kain KC, AJOG 2017
Copyright © 2017 Elsevier Terms and Conditions

## Slide 20
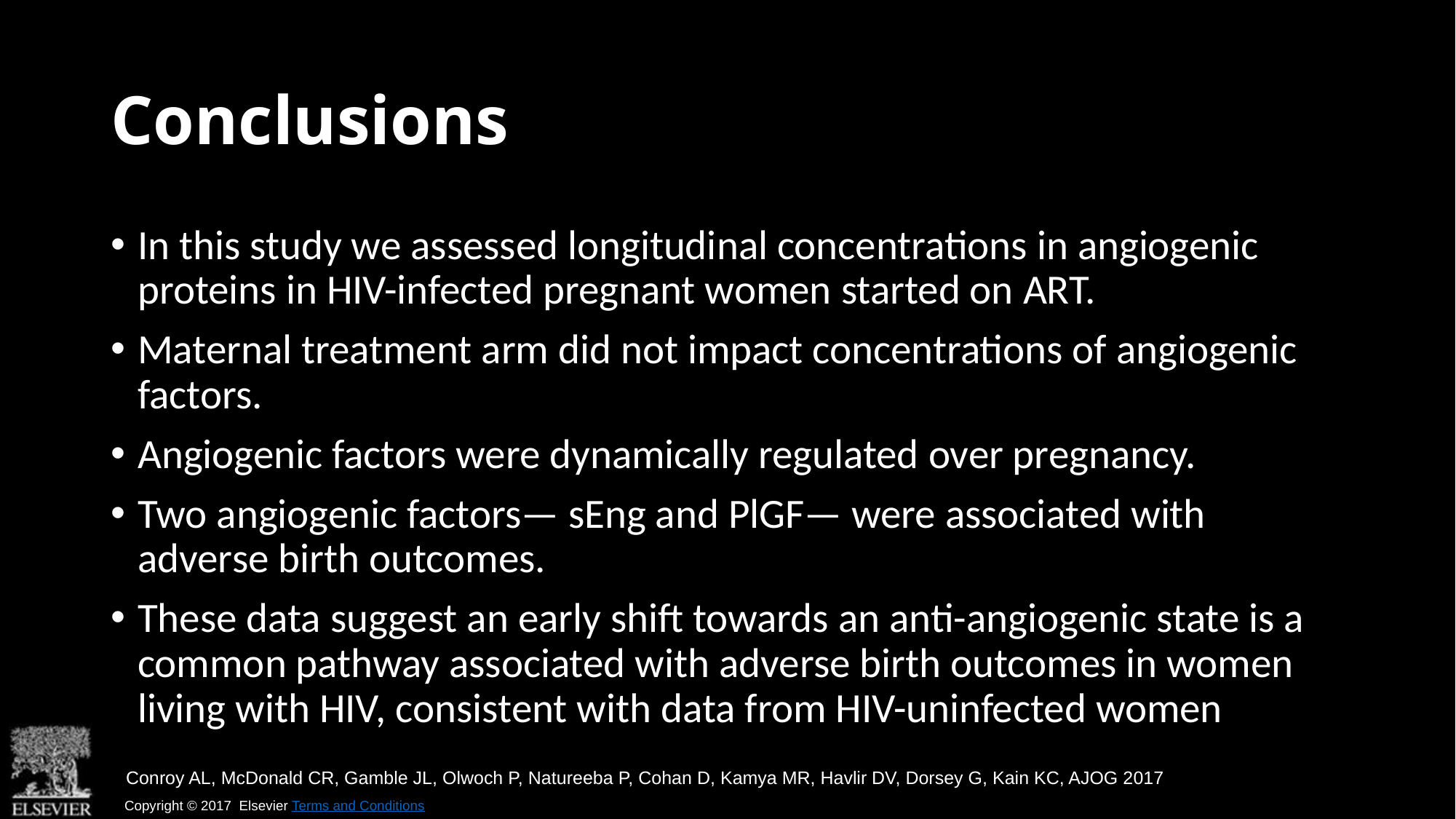

# Conclusions
In this study we assessed longitudinal concentrations in angiogenic proteins in HIV-infected pregnant women started on ART.
Maternal treatment arm did not impact concentrations of angiogenic factors.
Angiogenic factors were dynamically regulated over pregnancy.
Two angiogenic factors— sEng and PlGF— were associated with adverse birth outcomes.
These data suggest an early shift towards an anti-angiogenic state is a common pathway associated with adverse birth outcomes in women living with HIV, consistent with data from HIV-uninfected women
Conroy AL, McDonald CR, Gamble JL, Olwoch P, Natureeba P, Cohan D, Kamya MR, Havlir DV, Dorsey G, Kain KC, AJOG 2017
Copyright © 2017 Elsevier Terms and Conditions
